# Graph-Based Multi-Omics Integration Reveals Prognostic Histone Modification Reader Genes and Candidate Drug Targets in Colorectal Cancer

**DOI:** 10.3390/genes17080848

**Published:** 2026-07-23

**Authors:** Xiangjun Cui, Sibo Xue, Peijun Jiang, Langlang Shi, Tianyang Tan, Yuhan Xu, Guoqing Liu, Hu Meng, Guojun Liu, Yongqiang Xing

**Affiliations:** 1 School of Life Science and Technology, Inner Mongolia University of Science and Technology, Baotou 014010, China; cuixj@imust.edu.cn (X.C.);; 2 Inner Mongolia Key Laboratory of Life Health and Bioinformatics, Inner Mongolia University of Science and Technology, Baotou 014010, China

**Keywords:** multi-omics, histone modification reader, graph attention network, immune microenvironment, colorectal cancer

## Abstract

Background: Colorectal cancer (CRC) is driven by genetic alterations, epigenetic dysregulation and tumor microenvironment remodeling. Histone modification reader proteins serve as key epigenetic regulators of anti-tumor immunity, yet their synergistic immune networks, combined prognostic roles and immune subtype heterogeneity remain poorly understood. Methods: Here, we integrated multi-omics data and graph attention networks (GAT) to systematically screen prognostic-associated histone reader genes. We then conducted analyses using the immunoassay pipeline and ultimately identified hub immune-related genes validated in independent external cohorts. Additional analyses, including single-cell RNA sequencing (scRNA-seq), molecular docking and multiple in silico functional assays, were performed based on retrospective public datasets. Results: Four core genes (*CUL7*, *GPC1*, *NFYA*, *SLC25A5*) exhibited robust prognostic performance and strong correlations with anti-tumor immunity. Both core and auxiliary genes participate in critical metabolic and immune pathways. Candidate drugs present differential binding affinity for their encoded proteins, with sapitinib designated as a promising agent. Conclusions: This work constructs an epigenetic immune regulatory network and a four-gene signature, offering promising biomarkers and actionable therapeutic targets for precision immunotherapy against CRC.

## 1. Introduction

CRC is a leading cause of cancer mortality worldwide, driven by genetic mutations, epigenetic dysregulation, and tumor microenvironment (TME) remodeling [1,2]. Although targeted therapy and immunotherapy have advanced substantially, epigenetic heterogeneity and acquired drug resistance still markedly limit clinical outcomes. It is therefore urgent to explore epigenetic regulation, especially reversible histone modifications, as promising targets for molecular subtyping and immunotherapeutic response prediction [3].

Histone modification reader proteins interpret distinct histone marks to remodel chromatin and control gene transcription, forming a sophisticated epigenetic regulatory network [4]. Decoding histone mark–reader–target gene axes is critical for revealing CRC malignant traits and identifying actionable therapeutic targets. Readers function as core epigenetic sensors governing transcription, DNA repair and chromatin remodeling. However, their systematic regulatory patterns and crosstalk with the TME remain incompletely defined.

Mounting evidence indicates that dysregulated histone readers drive CRC progression. The Bromodomain and Extra-Terminal (BET) family, as classic acetylation readers, cooperates with mitogen-activated protein kinase (MAPK) inhibitors to suppress CRC by interfering with H3K27ac-dependent transcription, particularly in B-Raf proto-oncogene, serine/threonine kinase (BRAF) V600E-mutant tumors [5,6,7]. The YEATS (YAF9, ENL, AF9, TAF14, SAS5)-domain protein GAS41 recognizes H3K27ac and recruits BRD2–mediator complexes to promote CRC malignancy [8].

Methylation readers also play vital oncogenic roles. Plant homeodomain (PHD)-family proteins such as PHF14/PHF8 regulate DNA repair and epithelial–mesenchymal transition via H3K4me3 recognition [9,10]. Chromodomain proteins silence tumor suppressors through H3K27me3 [11]. Tudor and malignant brain tumor (MBT) domain proteins modulate oncogenic pathways and chromatin compaction to facilitate tumor growth [12,13,14]. Tryptophan-aspartic acid 40 (WD40)-repeat proteins including WDR5 maintain genome stability and chemoresistance [15,16,17]. The polycomb repressive complex 2 (PRC2) component embryonic ectoderm development (EED) reads H3K27me3 to repress transcription and modulate CRC immune microenvironments [18]. The ankyrin-containing methyltransferase G9a interprets H3K9 methylation to amplify oncogenic epigenetic signatures and correlate with poor recurrence-free survival [19,20].

Beyond acetylation and methylation, other reader families are also implicated in CRC. The 14-3-3 family modulates phosphatidylinositol 3-kinase (PI3K)/protein kinase B (AKT) signaling to constrain CRC migration and invasion [21]. The ubiquitin-binding E3 ligase RNF168 maintains genomic stability by recognizing histone ubiquitination. Its abnormal phase separation impairs DNA repair and drives chemoresistance in CRC [22,23,24]. The MACRO (mono-ADP-ribose-binding)-domain histone variant macroH2A1.1 binds ADP-ribose moieties, with isoform expression strongly correlated with CRC clinical prognosis [25].

Notably, histone readers exert nonlinear regulatory effects and extensive crosstalk within epigenetic networks. Identical histone marks can be recognized by distinct readers and consequently induce opposite biological phenotypes, making systematic profiling of synergistic gene networks indispensable [10]. Combinatorial histone marks jointly govern gene transcription. For instance, concurrent activation of H3K4me3 and H3K27ac upregulates long intergenic non-protein coding RNA 1605 (LINC01605), which further strengthens methyltransferase like 3 (METTL3)-dependent m^6^A (N6-methyladenosine) modification to modulate immune responses [26,27]. Despite abundant findings on combined histone modification patterns, existing research predominantly centers on separate epigenetic molecules or discrete signaling cascades. Comprehensive integrative analyses that connect histone modification profiles with gene transcription and tumor immune microenvironment (TIME) remain scarce [28,29]. These multilayered regulatory events feature high dimensionality and nonlinear interactions, consisting of diverse chromatin regulators, multi-layered omics information and heterogeneous immune constituents [30,31,32]. Advanced deep learning algorithms are well suited to resolve such complicated multi-omics nonlinear relationships.

**Figure 1:**
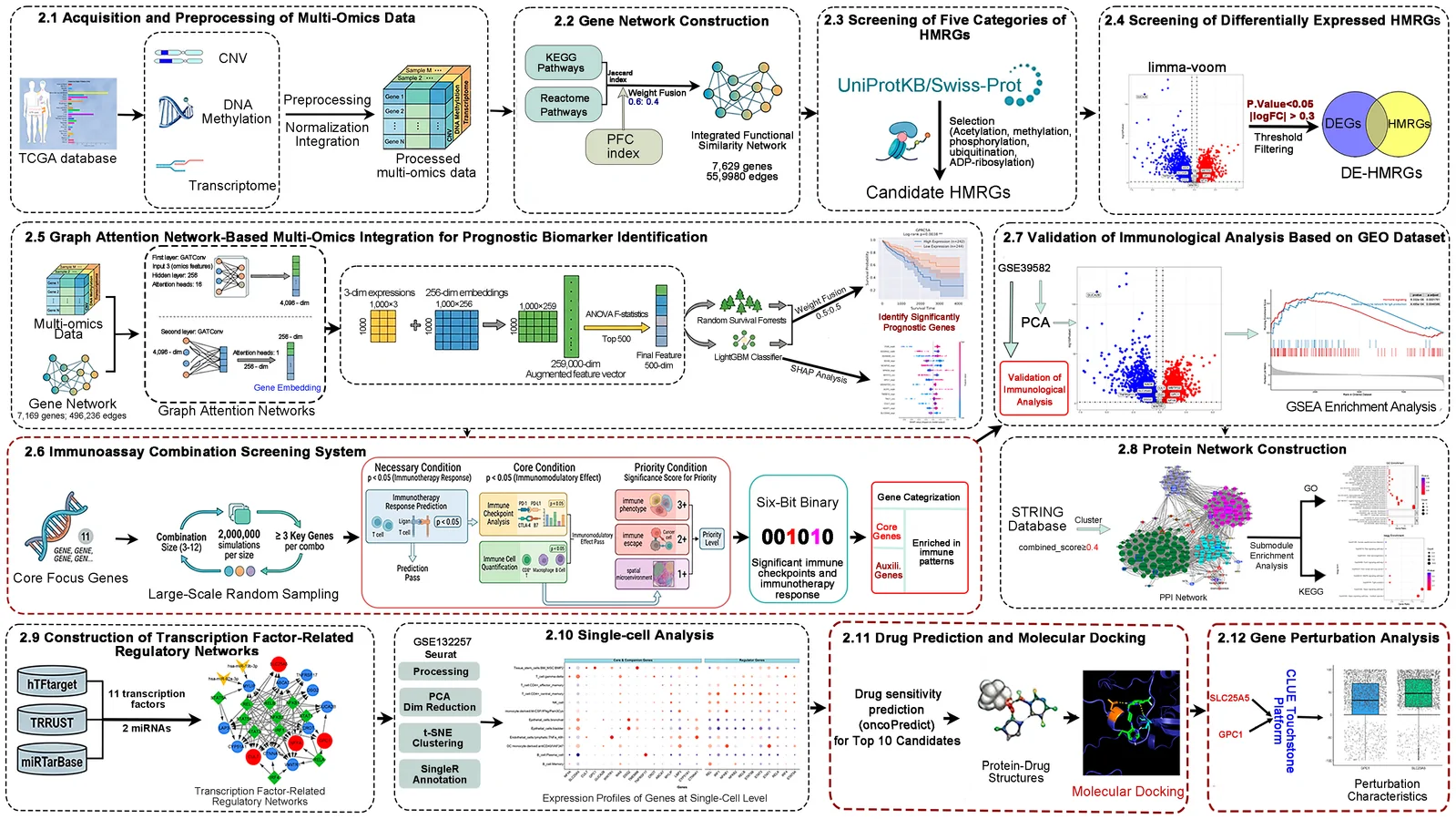
This schematic summarizes the complete analytical pipeline of this work, corresponding to Methods Section 2.1, Section 2.2, Section 2.3, Section 2.4, Section 2.5, Section 2.6, Section 2.7, Section 2.8, Section 2.9, Section 2.10, Section 2.11 and Section 2.12. Multi-omics profiles (CNV, DNA methylation, transcriptome) were retrieved from TCGA, followed by standardization, batch correction and grouping. Pathway annotations from KEGG and Reactome were integrated with the PFC index (weight ratio 0.6:0.4) to construct a gene functional similarity network containing 7629 genes and 559,980 edges. HMRGs were curated from UniProtKB/Swiss-Prot. Differentially expressed genes were identified via limma-voom (|log_2_FC| > 0.3, FDR < 0.05) and intersected with HMRGs. A two-layer GAT was used to integrate multi-omics data and gene topological information, generating 256-dimensional gene embeddings further refined into 500 representative features. Prognostic genes were screened using RSF, LightGBM, SHAP analysis and log-rank tests. Subsequent gene combination pattern and immune infiltration analyses enabled the definition of core and auxiliary genes. The GSE39582 cohort was used for independent validation via PCA, differential expression analysis and GSEA. We further constructed a PPI network, performed Leiden clustering and GO/KEGG enrichment, and reconstructed hierarchical TF–miRNA regulatory networks. Single-cell transcriptomes (GSE132257) were used for cell-type annotation and AUCell-based gene set activity quantification. Finally, candidate therapeutic compounds were predicted, and molecular docking was implemented for prioritization. In silico perturbation was performed to validate the functional roles of core genes.

Here, we constructed a 7629-gene functional similarity network using The Cancer Genome Atlas (TCGA) multi-omics data. Histone modification reader genes (HMRGs) were retrieved from UniProtKB/Swiss-Prot, and tumor-associated differentially expressed histone modification reader genes (DE-HMRGs) were filtered. A two-layer GAT extracted topological and multi-omics embedding features. Integrating random survival forest (RSF), Light Gradient Boosting Machine (LightGBM), and SHapley Additive exPlanations (SHAP) and Kaplan–Meier analysis, four core prognostic HMRGs were validated in Gene Expression Omnibus (GEO) cohort GSE39582. Optimized models defined five immune subtypes and generated a core HMRG-based epigenetic subtype signature (Figure 1).

## 2. Materials and Methods

### 2.1. Multi-Omics Data Acquisition and Preprocessing

CRC multi-omics data were retrieved from the TCGA database (https://www.cancer.gov/tcga (accessed on 28 July 2025)), including transcriptome (517 samples), DNA methylation (650 samples), and copy number variation (CNV) data (1547 samples). Methylation probes were annotated and aggregated to gene level using the biomaRt R package (version 2.66.2) [33,34]. Transcriptome raw counts were converted to Transcripts Per Million (TPM) and processed with Z-score normalization. DNA methylation data were also standardized by Z-score. Raw CNV values were kept without normalization to preserve the biological meaning of amplification and deletion alterations, with only sample matching and gene annotation conducted.

Following TCGA naming criteria, “01A” samples were defined as primary tumors and “11A” as adjacent normal tissues to establish separate tumor and normal expression matrices, control tissue heterogeneity, and guarantee subsequent multi-omics analysis reliability.

### 2.2. Gene Association Network Construction

To construct a genome-wide gene functional similarity network, we first calculated pathway-based pairwise similarity via the Jaccard index separately from Kyoto Encyclopedia of Genes and Genomes (KEGG) and Reactome pathway resources. The two sets of Jaccard coefficients were averaged under an equal 0.5:0.5 weighting to produce consolidated pathway similarity scores after normalization and reciprocal supplementation of missing values. The pathways containing fewer than five constituent genes were discarded to filter low-quality background noise. Following ENTREZ-to-ENSG identifier conversion, the integrated pathway similarity matrix was further fused with the protein functional coupling (PFC) index at a predefined 0.4:0.6 weight ratio [35]. For gene pairs without available PFC records, final similarity scores exclusively depended on consolidated pathway similarity values. The PFC metric aggregates multi-source evidence including co-expression, physical protein binding, genetic association and conserved protein domain interaction to depict holistic functional associations, whereas Jaccard similarity solely originates from incomplete pathway annotation datasets, justifying its lower assigned weighting in the composite calculation.

Sensitivity analysis using three weight ratios (0.6:0.4, 0.5:0.5, 0.4:0.6) and Spearman correlation was performed to validate network robustness.

### 2.3. Screening of Five Categories of HMRGs

Proteins containing conserved histone modification-recognition domains were retrieved from the UniProtKB database via keyword search of characteristic domain sequences. We collected all proteins harboring domains that theoretically mediate recognition of five major histone post-translational modifications: acetylation (bromodomain, YEATS, and tandem PHD finger), methylation (chromodomain, Tudor, PHD finger, MBT, WD40 repeat, and ankyrin repeat), phosphorylation (14-3-3 family, BRCA1 C-terminal (BRCT), and baculovirus IAP repeat (BIR)), ubiquitination (ubiquitin-binding domains (UBDs)), and ADP-ribosylation (MACRO domain and poly (ADP-ribose) binding zinc-finger (PBZ)). All domain-containing proteins were retained as preliminary HMRG candidates without additional manual filtering to exclude proteins that solely bind non-histone substrates.

To guarantee the reliability of gene annotations, only manually curated entries from UniProtKB/Swiss-Prot were incorporated, while unreviewed UniProtKB/TrEMBL records were discarded to reduce annotation errors and redundant entries. Subsequent differential expression, multi-omics integration, survival, and immune infiltration analyses were performed to filter candidate genes with functional roles in CRC and tumor immune microenvironment remodeling.

### 2.4. Screening of Differentially Expressed HMRGs

Differential expression analysis between tumor and normal samples was performed using the standard limma-voom workflow in R [36]. Genes with adjusted *p*-value (adj.*p*-value) < 0.05 and |log_2_ fold change (log_2_FC)| > 0.3 were considered significantly differentially expressed. The differentially expressed genes (DEGs) were further intersected with the identified HMRGs to obtain final DE-HMRGs for subsequent analyses.

### 2.5. GAT-Based Multi-Omics Integration for Prognostic Biomarker Identification

All models were implemented in Python 3.9. RSF was constructed using scikit-survival 0.22.0. LightGBM 4.0.0 was applied for classification. GAT was built with PyTorch Geometric 2.2.0. Model interpretability was analyzed via SHAP 0.41.0 [37,38,39,40].

#### 2.5.1. Data Sources and Preprocessing

Six datasets were integrated: three-dimensional multi-omics matrix consisting of 17,395 genes across 411 samples, gene annotation, clinical metadata, a co-expression network containing 559,980 edges, 7629 DEGs (|log_2_FC| > 0.3, False Discovery Rate (FDR) < 0.05), and survival information of 411 patients. After removing missing values, genes shared across multi-omics, network and DEG datasets were retained.

#### 2.5.2. Gene Network Construction and Feature Selection

The initial network contained 7629 genes. After omics data filtering, 7169 valid gene nodes and 496,236 weighted edges remained (correlation coefficient range: 0.061–1.000). Intersection of network nodes and DEGs yielded 1582 candidate genes. The top 1000 genes ranked by differential expression significance were selected for downstream modeling. The remaining genes were embedded via GAT to supplement topological information.

#### 2.5.3. Enhanced GAT Architecture

A two-layer GAT framework was constructed. The first layer was configured with 3 input channels, 256 hidden units, and 16 attention heads, with a dropout rate of 0.2. Batch normalization was applied between the two layers. The second layer, a single-head attention layer, aggregated features and output 256-dimensional gene embeddings to integrate multi-omics data and topological features.

Furthermore, we set the number of attention heads to 4, 8, 16, and 32 respectively, and compared the performance of the models under different settings.

#### 2.5.4. Node Feature Construction

For 7169 gene nodes, outlier filtering (mean ± 3SD) was performed per omics profile, and median expression was taken as the representative value. Transcriptome and methylation data were Z-score standardized. CNV data remained in raw scale to retain biological alteration information. Finally, a 7169 × 3 standardized node feature matrix was constructed.

#### 2.5.5. Feature Enhancement and Dimensionality Reduction

Each gene was represented by a fused 259-dimensional feature (3 original omics features + 256 GAT embeddings). For each sample, 1000 gene features were concatenated to form a 259,000-dimensional profile. Analysis of Variance (ANOVA) F-statistic-based supervised selection was used to retain the top 500 survival-related features for subsequent modeling.

#### 2.5.6. Machine Learning Model Construction and Evaluation

The top 500 filtered features were imported to construct RSF and LightGBM prognostic models. For RSF, samples were randomly divided into training and testing subsets at a 7:3 ratio, and the concordance index (C-index) served as the primary evaluation metric with empirically tuned hyperparameters. LightGBM was validated via five-fold stratified cross-validation with appropriate regularization and early stopping strategies, and model discrimination was quantified by Area Under the Receiver Operating Characteristic Curve (AUC-ROC). Final gene ranking was determined by averaging feature importance scores derived from the two independent algorithms.

#### 2.5.7. Model Interpretability Analysis

SHAP analysis was applied to interpret the optimal LightGBM model. Feature contributions were quantified and combined with RSF importance to screen core prognostic genes.

#### 2.5.8. Survival Analysis and Prognostic Gene Screening

Log-rank tests were performed on 1000 candidate genes to evaluate their prognostic significance. Patients were stratified into high- and low-expression subgroups based on the median gene expression. To guarantee statistical robustness, subgroups containing fewer than five patients were excluded. Genes with a log-rank *p*-value < 0.05 were considered prognostically significant.

### 2.6. Gene-Embedded Immunoassay Combination Screening System

This embedding-based screening framework comprises two interdependent modules: gene filtering and immune phenotype analysis. Joint execution of the two modules enables comprehensive and precise immune profiling.

#### 2.6.1. Gene Data Screening

From the 1000 candidate genes, the top 500 with the most significant log-rank prognostic outcomes (Section 2.5.8) were retained. Their 259-dimensional composite features were extracted as core inputs for downstream combinatorial immune screening.

#### 2.6.2. Immunoassay Pipeline

Based on the aforementioned log-rank prognostic screening results, prognostically significant DE-HMRGs were defined. Stratified random sampling (2 million replicates per combination size) was used to produce gene combinations of 3–12 members, with each assembly containing a minimum of three prognostically significant DE-HMRGs. We established a six-dimensional evaluation system covering immunotherapeutic response, immune checkpoint status, immune cell abundance, immune phenotype, immune escape, and TIME spatial features. Null *p*-values were simulated from a Beta distribution (*α* = 2, *β* = 8), and fixed random seeds were set to guarantee computational reproducibility. Beta distribution fits *p*-values bounded between 0 and 1 and accommodates the skewed distribution of our immune metrics, unlike normal, gamma or uniform distributions.

A three-tier screening criterion was applied: I. Significant correlation with immunotherapeutic response (*p* < 0.05); II. Significant difference in immune checkpoint or immune cell abundance (*p* < 0.05); III. Priority ranked by the number of significant items in the remaining three dimensions.

Each gene combination was assigned a six-digit binary signature, with 1 representing significance (*p* < 0.05) and 0 standing for non-significance. Five distinct immunological response patterns were classified according to these binary codes:

Pattern 1 (001010): significant in immune checkpoints and immunotherapeutic response.

Pattern 2 (100010): significant in immune cell abundance and immunotherapeutic response.

Pattern 3 (001110): significant in immune checkpoints, immune escape, and immunotherapeutic response.

Pattern 4 (011010): significant in immune phenotypes, immune checkpoints, and immunotherapeutic response.

Pattern 5 (100110): significant in immune cell abundance, immune escape, and immunotherapeutic response.

Genes enriched in the above immune response patterns were classified into core and auxiliary genes based on occurrence frequency and immune functional relevance.

### 2.7. External Validation Using Independent GEO Cohort

#### 2.7.1. GEO Data Organization and Differential Expression Analysis

The GSE39582 dataset from the GEO database (https://www.ncbi.nlm.nih.gov/geo/ (accessed on 29 December 2025)) was used for external validation, containing mRNA profiles of 566 CRC and 19 normal tissues. Probe annotation was performed using the hgu133plus2.db R package based on the GPL570 platform [41], and multiple probes corresponding to the same gene were averaged to generate an expression matrix comprising 20,824 unique genes. Principal component analysis (PCA) was used to assess transcriptional separation between tumor and normal tissues. Differential expression was calculated using the limma R package with cutoff criteria: |log_2_FC| > 0.3 and FDR < 0.05.

#### 2.7.2. Functional Enrichment Analysis of High-Frequency Auxiliary Genes

Gene Set Enrichment Analysis (GSEA) was performed on merged GEO-derived DEGs using the KEGG pathway database. Ensembl IDs were converted to Entrez IDs, and redundant genes were removed. Genes were ranked by descending log_2_FC for GSEA input. Enriched pathways were further filtered to retain those associated with core and auxiliary genes, clarifying their functional relevance to CRC progression.

#### 2.7.3. Validation of Immunological Analysis Based on GEO Dataset

The six-dimensional immunological analytical workflow in Section 2.6.2 was replicated in the independent GSE39582 cohort to validate the robustness of TCGA-derived immune patterns. All analytical pipelines and hyperparameters remained consistent with the TCGA analysis to avoid methodological bias. Cross-cohort reproducibility was evaluated by comparing immune signature distribution, core/auxiliary gene composition, and enriched pathway consistency between the two datasets.

### 2.8. Protein Network Construction

We constructed a protein–protein interaction (PPI) network for core and auxiliary genes via the STRING database [42], retaining interactions with a combined score ≥ 0.4. The network contained proteins encoded by candidate genes and their interacting partners. The network was visualized in Cytoscape 3.9 [43], and the Leiden algorithm was used for module detection using the clusterMaker2 plugin [44]. Gene Ontology (GO) and KEGG enrichment analyses based on hypergeometric tests were performed for each module, with corrected *p* < 0.05 defined as statistically significant.

### 2.9. Construction of TF-Related Regulatory Network

We used the hTFtarget database [45] to screen transcription factors (TFs) regulating core and auxiliary genes, and identified 11 key TFs: REL, IRF1, NFKB1, NFKB2, RELB, STAT5B, STAT3, STAT1, RELA, IRF4 and STAT5A. TF–gene interactions were validated using the TRRUST database. Target miRNAs were further retrieved from miRTarBase [46] to construct an integrated regulatory network comprising genes, TFs and miRNAs.

### 2.10. Single-Cell Transcriptomic Profiling

scRNA-seq data (GSE132257) were processed using the Seurat R package (version 5.2.1) with standard filtering and normalization for quality control [47,48]. Highly variable genes were retained, followed by PCA dimensionality reduction and t-distributed stochastic neighbor embedding (t-SNE) clustering. Cell clusters were annotated via SingleR (version 2.2.0) and celldex (version 1.10.1) based on the Human Primary Cell Atlas reference [49]. The AUCell package (version 1.22.0) was used to calculate single-cell area under the curve (AUC) scores for core genes, auxiliary genes and TF-related gene signatures [50,51]. Higher AUC scores indicate stronger signature expression and enhanced functional activity in individual cells. The resulting normalized AUC values were summarized in a dot heatmap, where dot diameter reflects the percentage of signature-positive cells per subset and color intensity represents Z-score-transformed normalized AUC levels.

### 2.11. Drug Prediction and Molecular Docking

#### 2.11.1. Drug Response Prediction

Drug sensitivity prediction of CRC patients was conducted via the oncoPredict R package (version 1.2) [52]. The model was trained on Genomics of Drug Sensitivity in Cancer phase 2 (GDSC2) cell line AUC profiles, and the full normalized transcriptome matrix covering all 17,395 genes from the 411 TCGA samples was used as the input dataset, consistent with the genome-wide input requirement of oncoPredict. Sample-specific drug response AUC scores were calculated with the calcPhenotype function. Lower AUC values represent stronger drug inhibitory effects on tumor cells. All compounds were sorted by AUC values in ascending order, and the top 10 drugs with the smallest AUC scores (highest predicted sensitivity) were selected for subsequent molecular docking validation. We further focused on candidate drugs showing sensitivity associated with the expression levels of the four core and eleven auxiliary CRC genes for targeted binding analysis.

#### 2.11.2. Molecular Docking Analysis

Molecular docking was performed to evaluate the binding affinity between candidate drugs and proteins encoded by four core genes (*CUL7*, *GPC1*, *NFYA*, *SLC25A5*) and 11 auxiliary genes (*GUCA2B*, *WWTR1*, *WAS*, *DSG2*, *TNFRSF17*, *CROT*, *ABCA7*, *MYLIP*, *LAP3*, *CYP51A1*, *CTNNA1*). The 15 target proteins were divided into three groups according to structural quality: Group 1 contained experimentally resolved proteins (SLC25A5, WAS, CTNNA1, LAP3, CYP51A1, CROT) with high-resolution three-dimensional (3D) structures (1.7–3.2 Å) obtained from the RCSB PDB database [53]. Group 2 included moderate-confidence AlphaFold models (GUCA2B, MYLIP, DSG2, CUL7, ABCA7) with per-residue local distance difference test (pLDDT) scores of 70–85, and regions with pLDDT ≥ 70 were used for docking [54]. Group 3 consisted of proteins with low-confidence structures (NFYA, GPC1, WWTR1, TNFRSF17), for which only structurally reliable functional domains were selected.

3D structures of candidate drugs were downloaded from PubChem in structure data file (SDF) format and converted to Protein Data Bank (PDB) format using Open Babel [55]. Protein and ligand preprocessing, including water removal, hydrogen addition, charge calculation, and protein data bank, partial charge and Q-type (PDBQT) format conversion, was conducted using AutoDockTools (version 1.5.6).

Semi-flexible docking was performed using AutoDock Vina. Docking boxes were defined based on predicted or known binding sites, with accuracy set to 8. Binding free energy (ΔG, kcal/mol) was used to evaluate binding affinity, with lower values indicating stronger interactions. Conformations with ΔG ≤ −7.0 kcal/mol were defined as strong binding. Optimal binding modes were visualized and analyzed using PyMOL (version 3.0) [56].

### 2.12. Gene Perturbation Analysis

In silico gene perturbation data were acquired from the Connectivity Map Linked User Environment (CLUE) Touchstone database (https://clue.io/touchstone (accessed on 7 January 2026)). The CRC cell line HT29 was used for perturbation analysis. Knockdown profiles of *SLC25A5* and *GPC1* were extracted via the Gene Knockdown module, with only HT29 datasets retained. Raw matrices were exported as TXT files. No knockdown data were available for *CUL7* and *NFYA* on this platform.

All data processing, statistics and visualization were completed using R. Perturbation effect scores were applied to assess transcriptomic changes after gene silencing. Box plots were used to compare perturbation magnitude between the two genes, where the middle line stands for the median, boxes for interquartile ranges and dots for individual values. Positive scores indicate upregulation of downstream transcripts, and higher scores represent stronger transcriptomic disturbance.

Analyses in Section 2.5 and Section 2.6 were performed using Python (version 3.9), and all other procedures were implemented using R software (version 4.5.2).

## 3. Results

### 3.1. Multi-Omics Data Integration

Multi-omics data were retrieved from TCGA. Three omics datasets were processed with probe annotation and gene aggregation. Common genes and samples shared across all omics modalities were intersected to generate an integrated expression matrix comprising 17,395 genes derived from 411 matched multi-omics samples.

A total of 517 raw transcriptomic samples were initially collected. After preliminary quality screening, 491 qualified samples were retained, including 447 tumor samples and 44 normal mucosal samples. Subsequent overlapping matching with DNA methylation and copy number variation (CNV) datasets yielded 411 samples with complete multi-omics profiles for integrated analysis.

### 3.2. Construction of the Gene Association Network

The gene association network was successfully constructed, encompassing a total of 7629 genes. Gene pairs with comprehensive functional similarity scores greater than zero were retained, resulting in a final network comprising 559,980 edges.

Sensitivity analysis revealed high correlations among the composite scores derived from three weighting schemes (0.6:0.4, 0.5:0.5, and 0.4:0.6), with Spearman correlation coefficients ranging from 0.995 to 0.999. Each ratio represents the weight assigned to PFC index versus Jaccard pathway similarity. This finding indicates that network construction is minimally dependent on the choice of weights, underscoring the robustness of the results. Relevant findings are presented in Figure S1.

### 3.3. HMRG Screening

Based on the UniProtKB/Swiss-Prot database, we systematically screened and classified CRC-related HMRGs. Phosphorylation-recognition HMRGs were the largest group, with 2169 genes in the 14-3-3 family. Ubiquitination-recognition HMRGs ranked second, containing 1201 genes with UBDs.

For methylation-recognition HMRGs, we identified 25 chromodomain, 91 Tudor domain, 294 PHD finger, 56 MBT domain, 256 WD40 repeat and 88 ankyrin repeat genes. Acetylation-recognition HMRGs included 52 bromodomain, 5 YEATS domain and 3 tandem PHD finger genes. ADP-ribosylation recognition category contained 16 macrodomain genes. The final HMRG set also included 30 BRCT and 15 BIR domain genes. All screened genes are listed in Table S1.

### 3.4. Screening of DE-HMRGs

Differential expression analysis was performed using the limma-voom method on transcriptomic data derived from tumor and normal tissue samples, with significance thresholds set at |log_2_FC| > 0.3 and adj.*p*-value < 0.05. A total of 9788 genes exhibiting significantly differential expression in CRC were identified. These DEGs were then intersected with the HMRGs listed in Table S1, ultimately yielding 1814 DE-HMRGs. Detailed information on these genes is provided in Supplementary Material Table S2.

### 3.5. GAT-Based Prognostic Gene Identification Analysis

#### 3.5.1. Gene Co-Expression Network

Following the successful integration of multi-omics data with gene network information, a robust co-expression network was constructed, encompassing 7169 valid gene nodes and 496,236 valid edges.

#### 3.5.2. Training Performance of the GAT

The GAT model was trained for 35 epochs with an early stopping strategy to prevent overfitting. As shown in Figure S2, training loss decreased steadily from 0.2937 to 0.1121 throughout the training process. Validation loss dropped rapidly initially and then fluctuated at a low level. The minimum validation loss appeared at the 15th epoch, which was selected as the optimal model checkpoint with the best generalization ability. After epoch 15, training loss continued to decline while validation loss slightly rebounded, indicating emerging overfitting and verifying the rationality of choosing the epoch 15 model.

A stepwise decay strategy was applied to the learning rate, starting at 0.001 and gradually reducing to 0.0005, 0.00025, and finally 0.00125. This strategy effectively ensured stable convergence and high optimization efficiency of the GAT model.

#### 3.5.3. Edge Weight Reconstruction Capability Evaluation

Pearson correlation analysis was used to evaluate the linear consistency between the GAT-learned gene embeddings and the original co-expression edge weights. We calculated the correlation between gene embedding-based cosine similarity and original network edge weights. A weak but highly significant positive correlation was observed (*r* = 0.201, *p* < 0.001, *R*^2^ = 0.040), suggesting that GAT only partially preserves intrinsic co-expression information during graph encoding. This limited linear consistency is also illustrated in Figure S3.

The GAT model does not merely replicate original edge weights during representation learning. Instead, learned gene embeddings integrate network topology and multi-omics node features to capture these nonlinear relationships, producing latent representations that complement and expand upon the initial co-expression relationships.

#### 3.5.4. Gene Embedding Clustering Visualization

t-SNE analysis showed that gene embeddings from the optimal GAT model exhibited clear functional clustering. Gaussian mixture model analysis classified genes into five clusters with 95% confidence ellipses delineating cluster boundaries (Figure S4).

Classic CRC driver genes such as *TP53*, *KRAS* and *EGFR* were distributed across different clusters, reflecting their diverse biological functions in CRC. The well-separated clustering further confirmed that the GAT model effectively captured gene functional similarities, demonstrating the reliability and advantages of the graph embedding approach.

#### 3.5.5. RSF Performance

The RSF model achieved a C-index of 0.9060 in the training cohort, but this dropped to 0.6530 in the test cohort (Figure S5), indicating overfitting. This performance decline can be largely attributed to the combination of high-dimensional gene features and a limited number of clinical samples, which increased model complexity and impaired its generalizability to external validation.

#### 3.5.6. LightGBM Cross-Validation Results

Following 5-fold stratified cross-validation, the LightGBM model yielded a mean AUC of 0.5451 ± 0.0714 (Figure S6). The AUC values across the five folds ranged from 0.4729 to 0.6806, with the best performance observed in the second fold (AUC = 0.6810). The corresponding ROC curve for this optimal fold (Figure S7) further suggests a modest discriminative potential for prognostic classification.

#### 3.5.7. Highly Consistent Feature Importance Rankings from RSF and LightGBM

Feature importance rankings generated by the RSF and LightGBM models exhibited an extremely strong positive Spearman correlation (*r* = 0.995, *p* < 0.001, Figure S8). Such a high consistency in key feature screening between two independent machine learning algorithms further confirms the robustness and reliability of the identified prognostic gene set.

#### 3.5.8. SHAP Interpretability Analysis

Three types of SHAP analyses were applied to interpret feature importance and regulatory patterns of the prognostic model (Figures S9–S11). Figure S9 displays the top 15 features ranked by mean absolute SHAP values, including multiple gene signatures and clinical indicators with diverse predictive contributions. The SHAP summary plot (Figure S10) illustrates the directional associations between key features and prognostic risk. Some genes were positively correlated with risk scores and poor prognosis, whereas others showed negative regulatory and protective effects, with obvious differences in impact magnitude. Figure S11 further compares gene predictive weights, revealing distinct disparities in prognostic contribution: several genes played dominant roles, while others served as auxiliary predictors.

#### 3.5.9. Screening of Prognostically Significant DE-HMRGs

Kaplan–Meier survival analysis with log-rank test was performed on 1000 candidate genes, and 11 DE-HMRGs with prognostic significance were finally identified in CRC patients (all *p* < 0.05).

**Figure 2:**
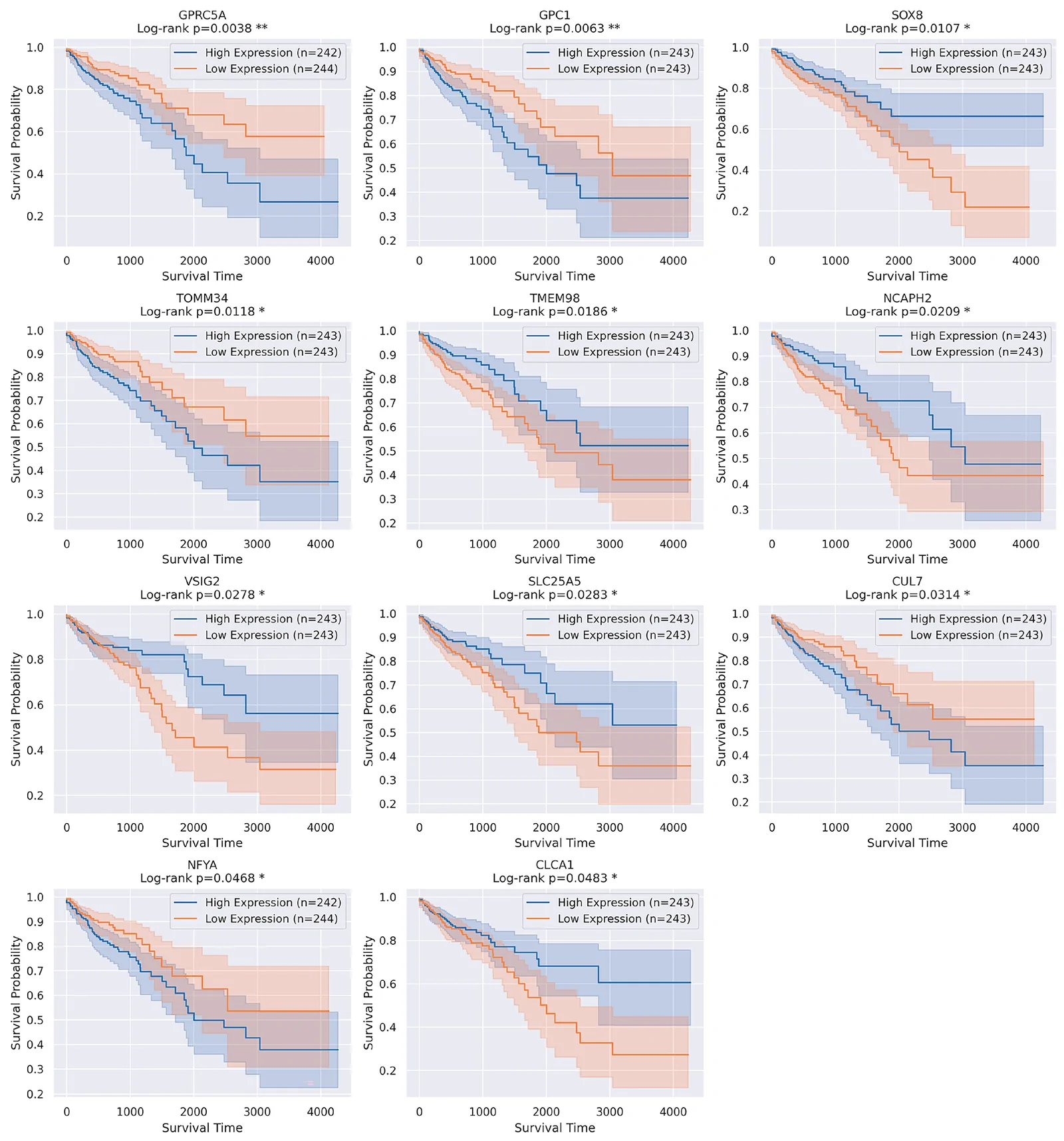
Kaplan–Meier survival analysis of 11 candidate genes. Y-axis, survival probability; X-axis, survival time. Blue curves, high-expression group; orange curves, low-expression group. Translucent shading indicates 95% confidence intervals. Gene symbols and log-rank *p*-values are labeled for each subplot; significance: *p* < 0.05 (*), *p* < 0.01 (**).

As shown in Figure 2, the 11 genes presented two distinct prognostic patterns. Five genes (*GPRC5A*, *GPC1*, *TOMM34*, *CUL7*, *NFYA*) acted as risk factors. Their high expression correlated with shorter overall survival. The other six genes (*SOX8*, *TMEM98*, *NCAPH2*, *VSIG2*, *SLC25A5*, *CLCA1*) served as protective factors, with higher expression associated with better prognosis. Among them, *GPRC5A* (*p* = 0.0038) and *GPC1* (*p* = 0.0063) reached *p* < 0.01, showing the strongest prognostic value, while the remaining genes had *p* values ranging from 0.0107 to 0.0483.

Figure S12 further showed 12 genes with marginally prognostic relevance (*p* value between 0.05 and 0.10). Seven genes including *ABCA7*, *YBX3*, *SLC2A3*, *RCN1*, *ALDH18A1*, *GGCT* and *ADAT1* tended to be poor prognostic factors, whereas another five genes (*SARS1*, *GUCA2B*, *PDK2*, *PDK4*, *IL17RB*) tended to be protective. *ABCA7* (*p* = 0.0567) and *YBX3* (*p* = 0.0569) exhibited the most obvious survival trends among these marginal candidates.

### 3.6. Analysis Results Based on Immunoassay Pipelines

#### 3.6.1. Model Complexity Comparison and Optimization

**Figure 3:**
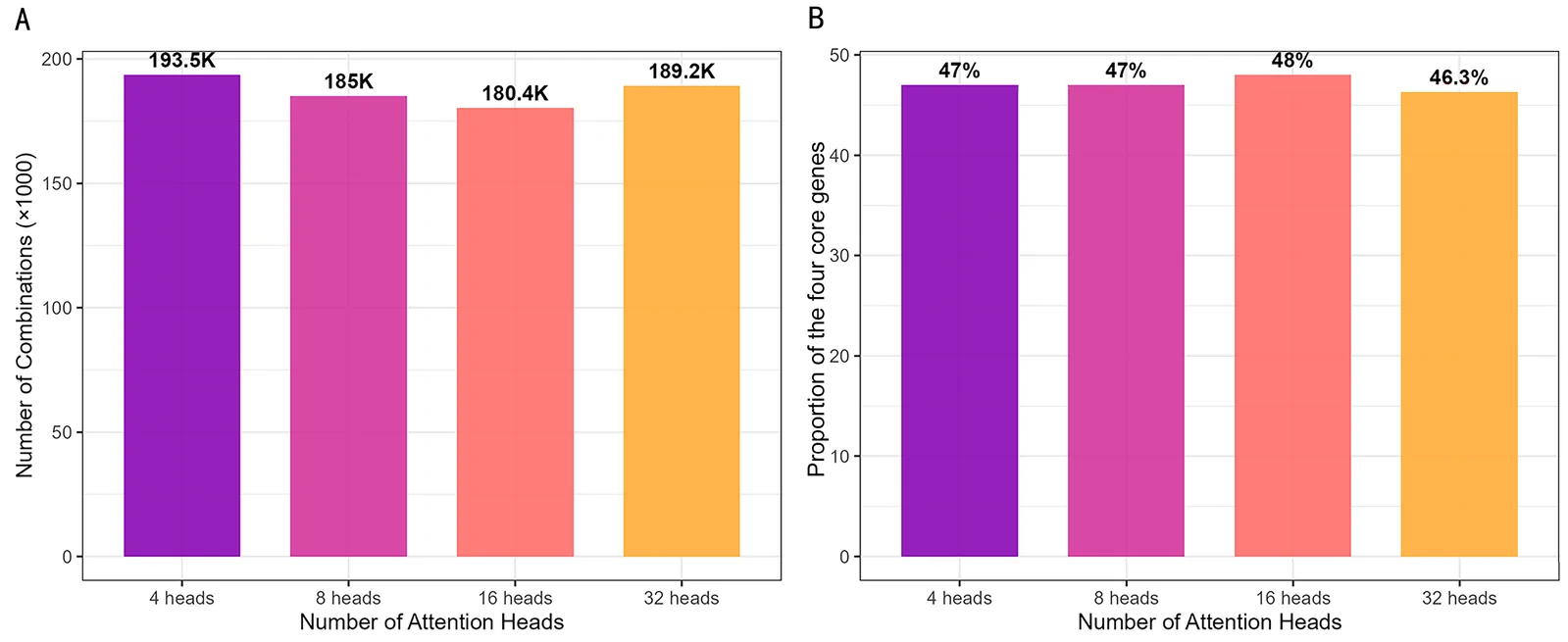
Overall performance and inherent characteristics of the prognostic models. (**A**) Counts of significant gene combinations across four integrated models. (**B**) Proportion of significant combinations containing the four core prognostic genes.

As shown in Figure 3A, the number of significant gene combinations exhibited a U-shaped trend with rising model complexity. From the 4-head to 16-head model, the count gradually decreased from 193,547 to 180,357—a total reduction of 6.8%. This decline suggested that moderate complexity suppressed noise and refined screening reliability. However, the 32-head model rebounded to 189,197, a 4.9% increase compared with the 16-head model. The U-shaped pattern indicated that the simple 4-head model retained redundant noise, while 8–16-head models achieved effective denoising and optimization. Excessive complexity in the 32-head model may introduce false correlations or capture unique biological signals overlooked by moderate-complexity models.

Figure 3B showed stable enrichment of the four core genes (which are illustrated in Figure 9A later) across all four models, with the 16-head model containing the highest proportion of core-gene combinations. The proportion of combinations covering all four core genes remained stable at 46–48% across models, verifying the robustness of core gene modules. The 16-head model reached the peak proportion of 48.0%, showing optimal ability to capture core functional modules, whereas the 32-head model slightly decreased to 46.3%.

**Figure 4:**
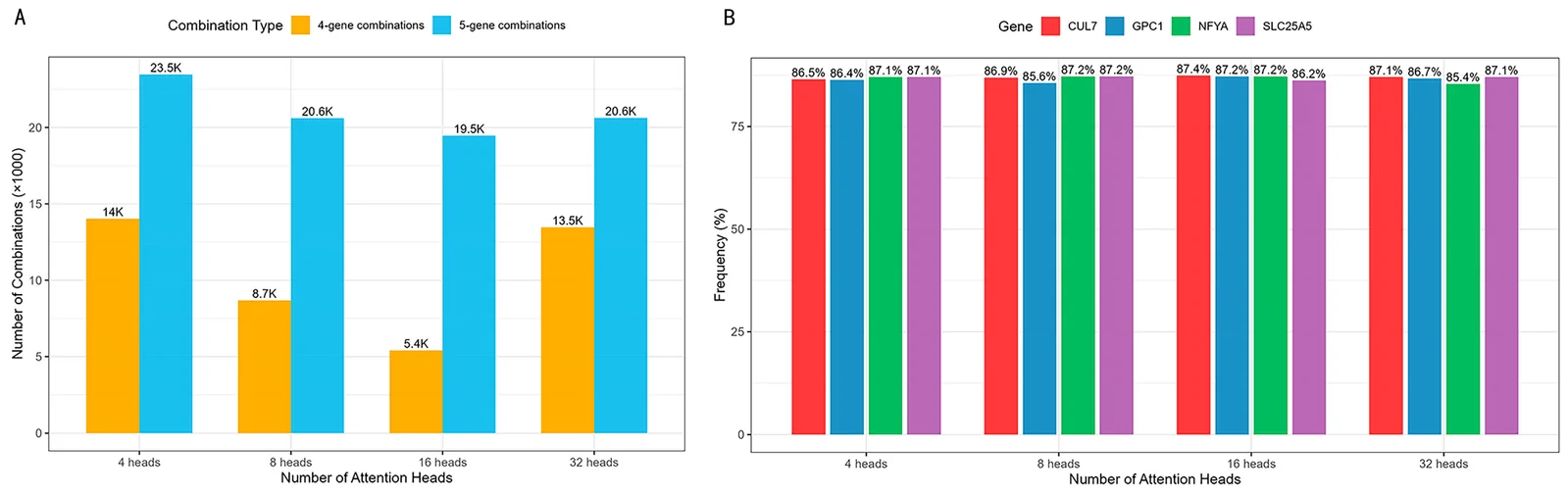
Variation in gene combination counts and core gene frequency across attention head configurations. (**A**) Number of significant small-scale gene combinations (4–5 genes). (**B**) Frequency of core genes under different attention head settings.

Figure 4A shows the filtering trends of small-scale gene combinations, consistent with the overall variation pattern. The number of 4-gene combinations dropped sharply from 14,027 (4-head) to 5407 (16-head, 61% reduction), then rebounded to 13,468 in the 32-head model. 5-gene combinations followed a milder similar trend, decreasing by 17% and then slightly recovering. The 16-head model exhibited the strictest screening for simple combinations, while the 32-head model retrieved more small-scale combinations.

Figure 4B depicts the frequency dynamics of four core genes across models. CUL7 peaked at 87.4% in the 16-head model. NFYA remained stable initially but declined markedly in the 32-head model. SLC25A5 maintained high and stable frequency, and GPC1 reached its highest proportion in the 16-head configuration. The 16-head model produced the most balanced frequency distribution of core genes with only a 1.2% amplitude difference.

**Figure 5:**
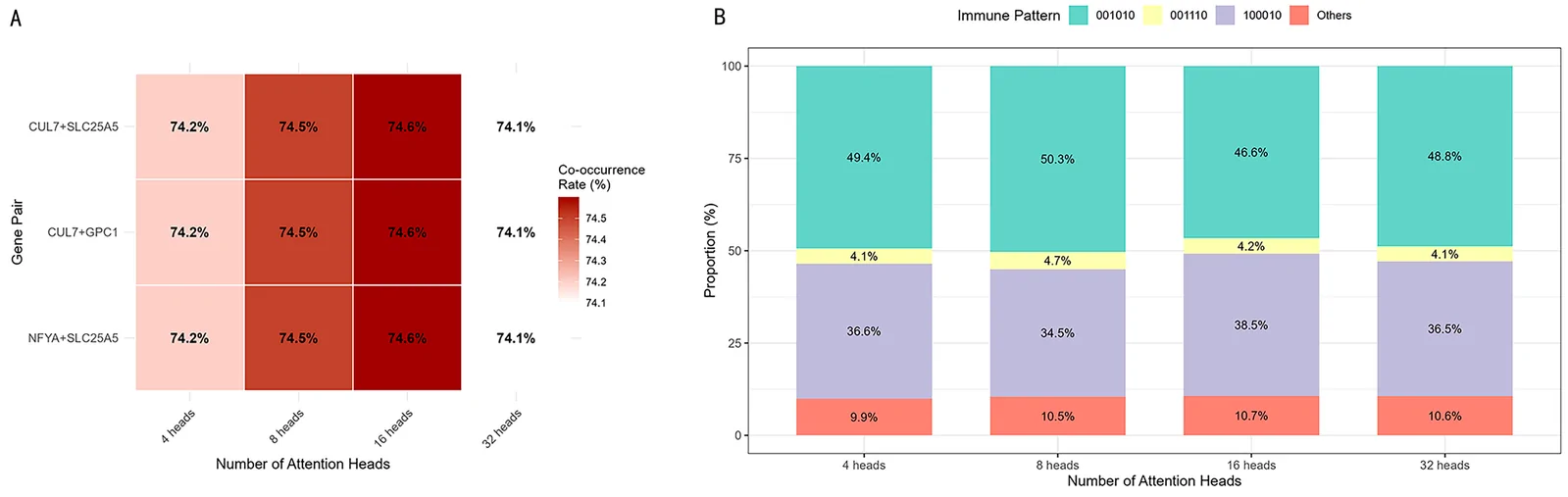
Co-occurrence and immune pattern distribution of core genes. (**A**) Co-occurrence pattern frequency of core genes. (**B**) Distribution of immune patterns.

Figure 5A shows the co-occurrence rates of the three most stable core gene pairs across different attention-head models. The pairs CUL7 + SLC25A5, CUL7 + GPC1, and NFYA + SLC25A5 maintained consistently high co-occurrence rates of 74.1–74.6% with minor fluctuations. Their rates gradually increased from the 4-head (74.2%) to the 16-head model (74.6%) and slightly decreased to 74.1% in the 32-head model, demonstrating stable cooperative relationships independent of model complexity. These findings suggest that the core gene pairs reflect intrinsic biological co-regulatory patterns in CRC rather than random correlations, validating their reliability as key prognostic signature components.

Figure 5B illustrates the distribution of immune patterns, which were generally conserved yet partially divergent across models. The two dominant patterns consistently accounted for over 80% of all profiles, indicating preserved core immune regulatory mechanisms. The 16-head model showed the most evident deviation: pattern 001010 decreased to 46.6%, while pattern 100010 peaked at 38.5%, suggesting an obvious shift in immune pattern composition. By comparison, the complex pattern 001110 remained stable at 4–5% and was insensitive to model configuration.

**Figure 6:**
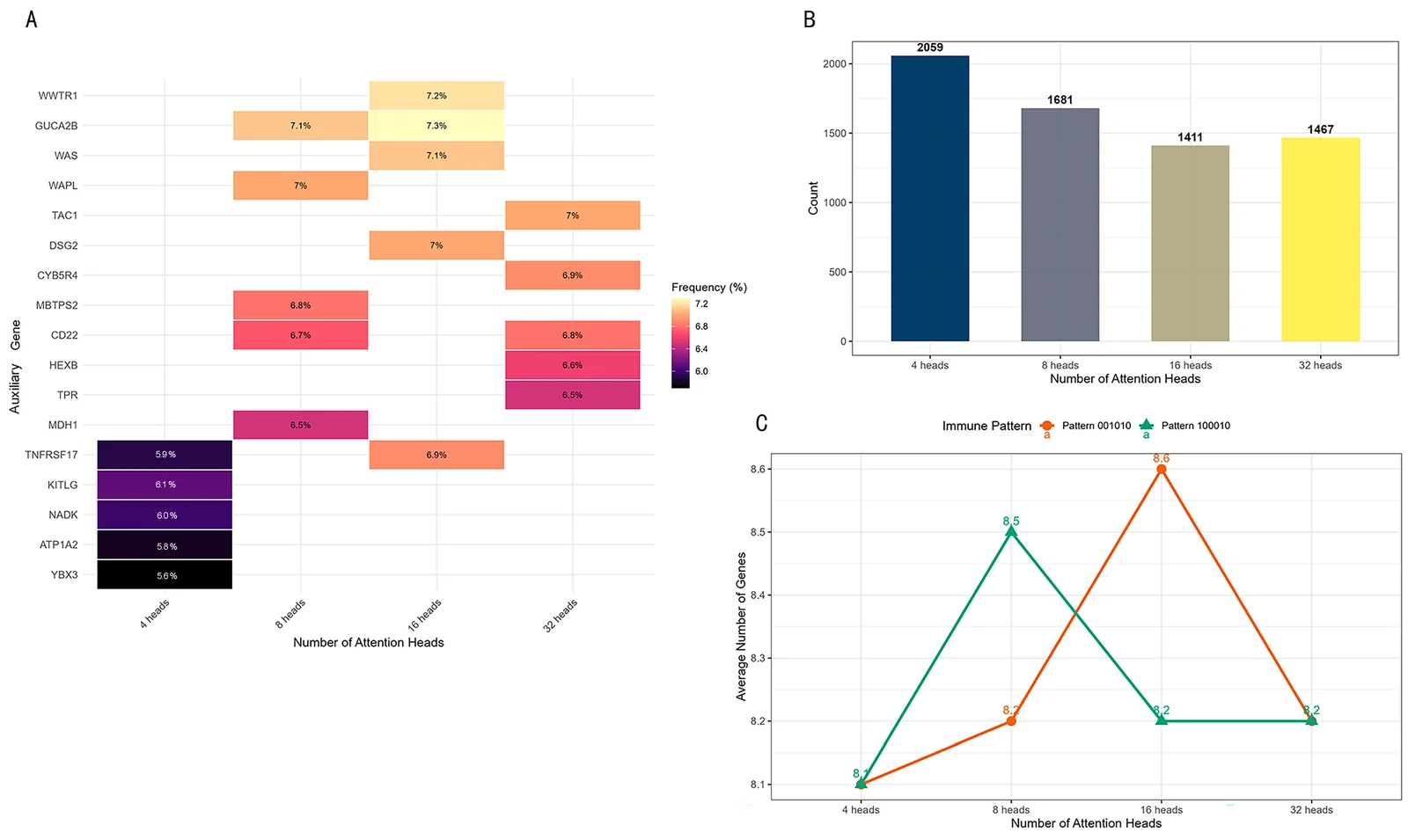
Auxiliary gene frequency and gene combination counts across immune patterns. (**A**) Frequency of top auxiliary genes across attention head configurations. (**B**) Number of significant combinations corresponding to Pattern 4 and Pattern 5. (**C**) Average gene number of gene combinations associated with Pattern 1 and Pattern 2.

Figure 6A heatmap shows obvious shifts in auxiliary gene sets (see Figure 9A) with increasing model complexity, with the 16-head model performing optimally. The 4-head model had low auxiliary gene frequencies (5.7–6.1%), while the 8-head model introduced new genes and raised frequencies to 6.5–7.1%. The 16-head model achieved the highest frequencies (6.9–7.3%) and the most stable composition. The 32-head model replaced auxiliary genes and slightly lowered frequencies to 6.5–7.0%. GUCA2B appeared in the 8-head and 16-head models but disappeared in the 32-head model, whereas CD22 existed in the 8-head and 32-head models, implying potential immunomodulatory roles.

Figure 6B presents the number of Pattern 4 and 5 combinations. The count steadily decreased from 2059 in the 4-head model to 1411 in the 16-head model (31% reduction) and slightly rebounded to 1467 in the 32-head model, confirming that the 16-head model adopted the strictest screening threshold.

Figure 6C depicts the dynamic average gene number per combination across model configurations. For Pattern 001010, the average gene count peaked at 8.6 in the 16-head model and dropped to 8.2 in the 32-head model. For Pattern 100010, the average peaked at 8.5 in the 8-head model and then declined, stabilizing at 8.2 in both the 16-head and 32-head models.

**Figure 7:**
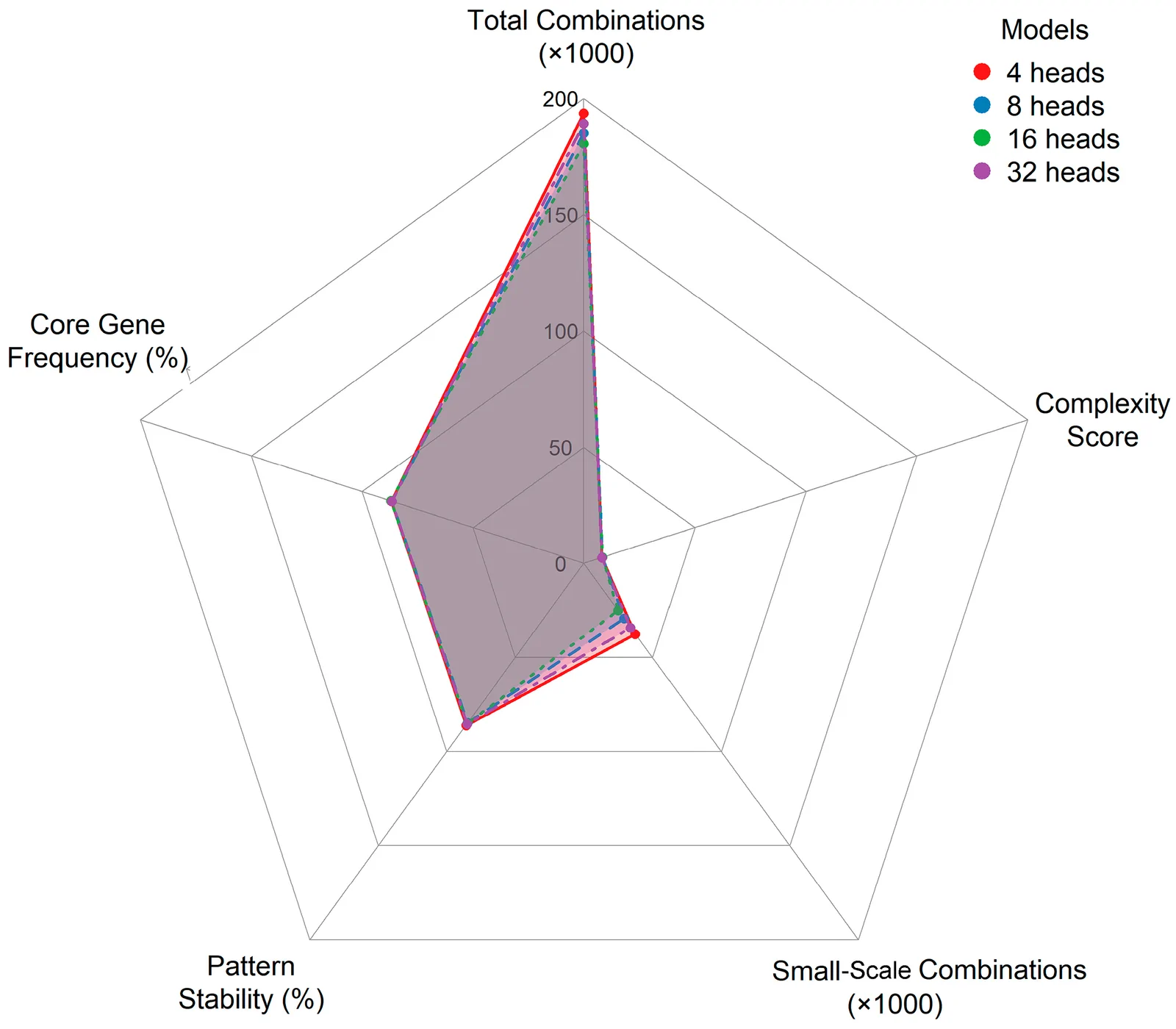
Model performance summary across different attention head configurations.

The radar chart in Figure 7 demonstrates the superior comprehensive performance of the 16-head model across five evaluation dimensions. It achieved optimal values in core gene frequency (87.0%), combination complexity (8.4, average gene number per combination), and simple combination screening (24.9K). With a total of 180.4K combinations, the 16-head model reached the best convergence and outperformed other configurations. The 8-head model showed acceptable but less prominent core gene features, while the 4-head and 32-head models obtained moderate scores due to excessive noise and potential overfitting, respectively.

**Figure 8:**
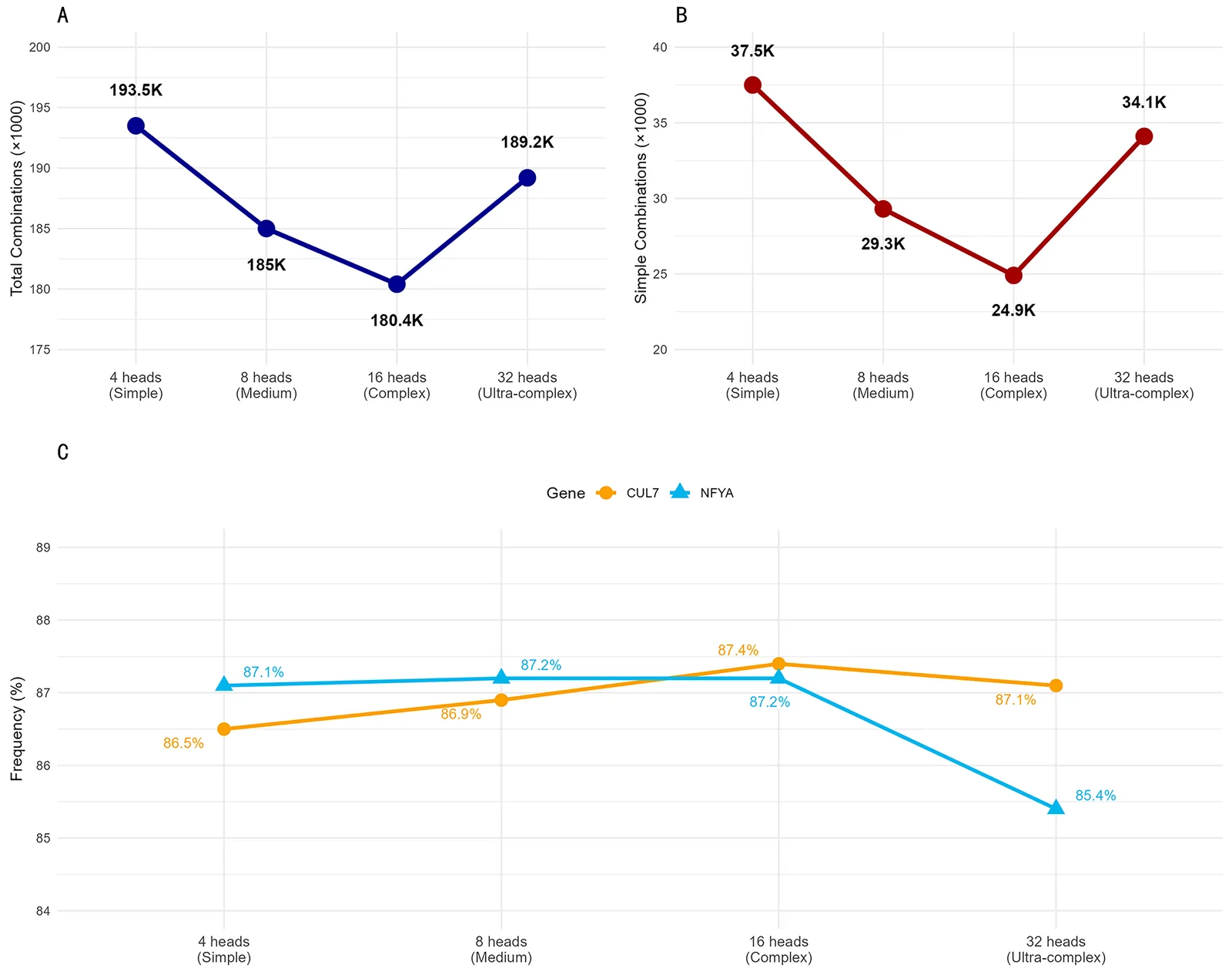
Feature combination and core gene dynamics dependent on attention head number. (**A**) Trends in total feature combination numbers across attention head settings. (**B**) Trends in simple feature combination numbers across attention head settings. (**C**) Dynamic frequency changes in core genes *CUL7* and *NFYA* with increasing attention heads.

Figure 8A–C show the evolutionary trends of three key metrics, with the 16-head model as the critical turning point. The total combination number decreased from 193.5 K to 180.4 K and then rebounded to 189.2K. The count of simple combinations declined from 37.5 K to 24.9 K before rising to 34.1K. For core gene dynamics, CUL7 peaked at 87.4% in the 16-head model, whereas NFYA began to drop after this point, decreasing from 87.2% to 85.4%. These trends indicate that the 16-head model represents a watershed: models underwent continuous refinement before this stage and gradually diverged afterwards.

Based on multiple evaluation indicators described above, the 16-head model was identified as the optimal framework. Its screening process achieved favorable convergence, generating a total of 180,357 combinations. Among them, CUL7 was the predominant core gene, accounting for 87.4%. This configuration delivered the strictest filtering for simple combinations, yielding 5407 four-gene clusters. It also maintained the most stable frequency of auxiliary genes (7.3%), balanced immune pattern distribution, and a reasonable average combination size ranging from 8.4 to 8.6 genes.

#### 3.6.2. Results of Immune Infiltration Analysis in a 16-Head Model

**Figure 9:**
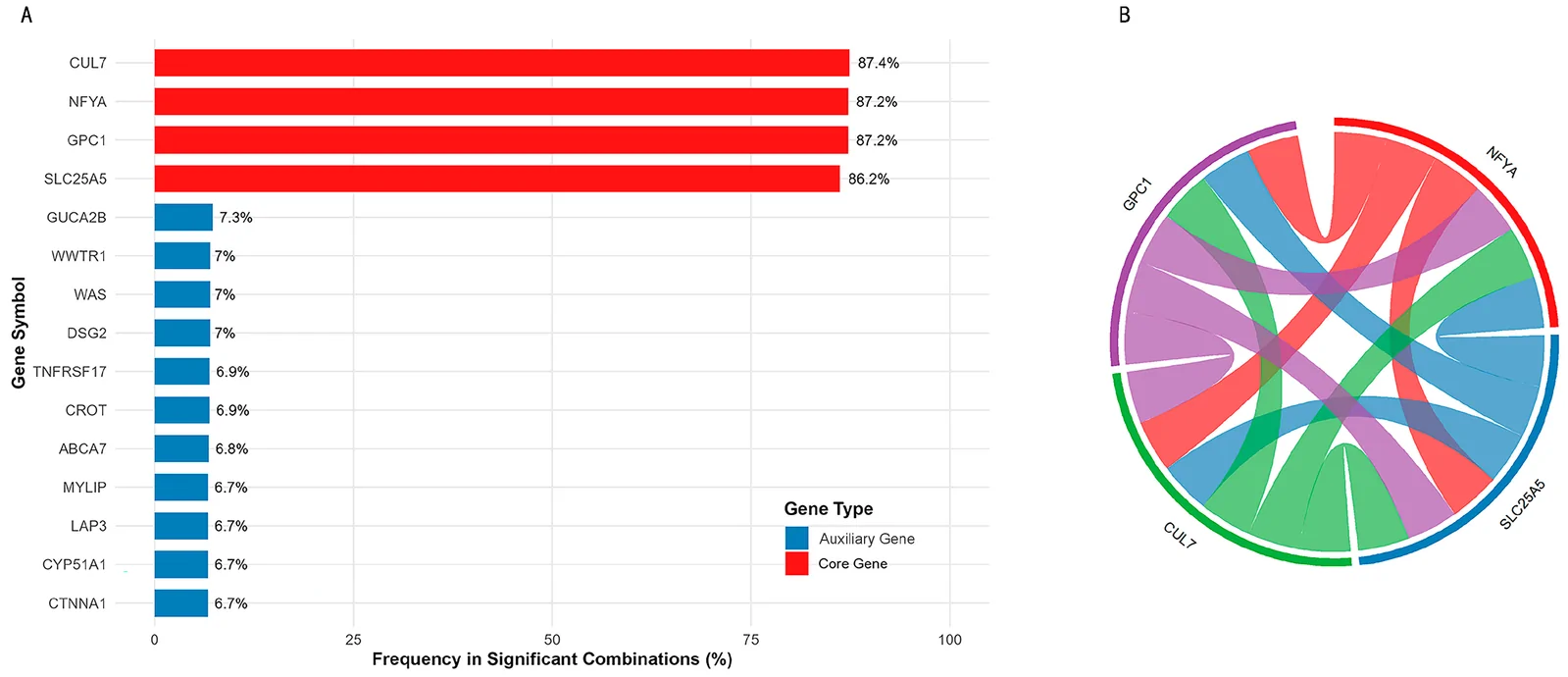
Frequency difference and co-occurrence network of core and auxiliary genes. (**A**) Frequency discrepancy between core and auxiliary genes in significant combinations. (**B**) Co-occurrence network of core genes.

Figure 9A illustrates the discontinuous frequency distribution of screened genes. These genes were classified into four core genes (*CUL7*, *NFYA*, *GPC1*, *SLC25A5*) and eleven auxiliary genes. The four core genes showed a significant correlation with CRC prognosis. Of the auxiliary genes, *GUCA2B* and *ABCA7* had marginal prognostic relevance, and the remaining nine genes (*WWTR1*, *WAS*, *DSG2*, *TNFRSF17*, *CROT*, *MYLIP*, *LAP3*, *CTNNA1*) showed no statistically significant prognostic associations.

Core genes had frequencies of 86.2–87.4%, markedly higher than auxiliary genes (6.9–7.3%, topped by GUCA2B). These data verify a stable regulatory module centered on the four core genes, with auxiliary genes serving as secondary modulators.

The STRING plot (Figure 9B) displays tight synergistic connections among core genes. The CUL7–GPC1 pair had the highest co-occurrence (74.6%), followed by CUL7–NFYA (74.5%) and NFYA–GPC1 (74.3%). SLC25A5 maintained balanced pairing rates (73.4–73.6%) with all partners, suggestive of a hub role. The 16-head model precisely captured these intrinsic synergistic interactions.

**Figure 10:**
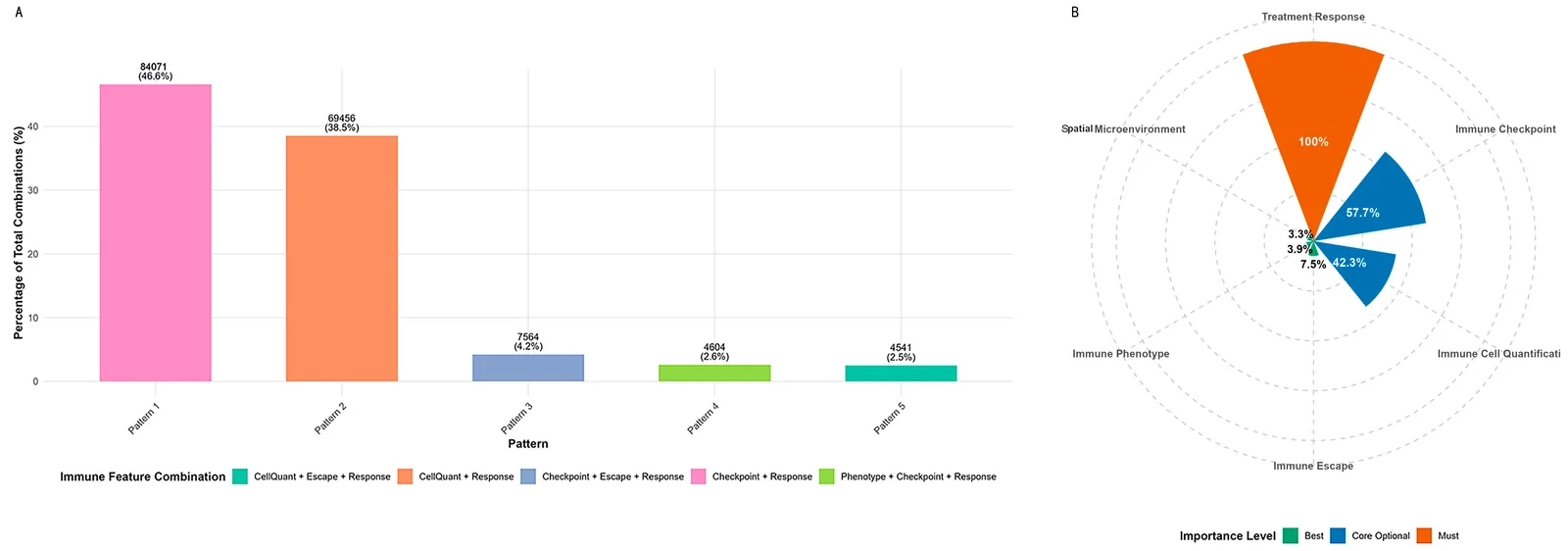
Distribution and significance frequency of immune patterns. (**A**) Distribution of classified immune analysis patterns. (**B**) Significance frequency of individual immune features.

Figure 10A quantifies five immune patterns. Pattern 1 (Checkpoint + Response, 46.6%) and Pattern 2 (CellQuant + Response, 38.5%) together occupied >85% of combinations and dominated core immune regulation. Patterns 3–5 only accounted for 2.5–4.2% and corresponded to complex immune configurations.

The polar plot (Figure 10B) reveals hierarchical immune features: treatment response was universal (100%), immune checkpoint (55.0%) and cell quantification (45.0%) were core optional markers, whereas immune escape, phenotype and spatial microenvironment (3.3–7.5%) were rare fine-tuning indicators.

**Figure 11:**
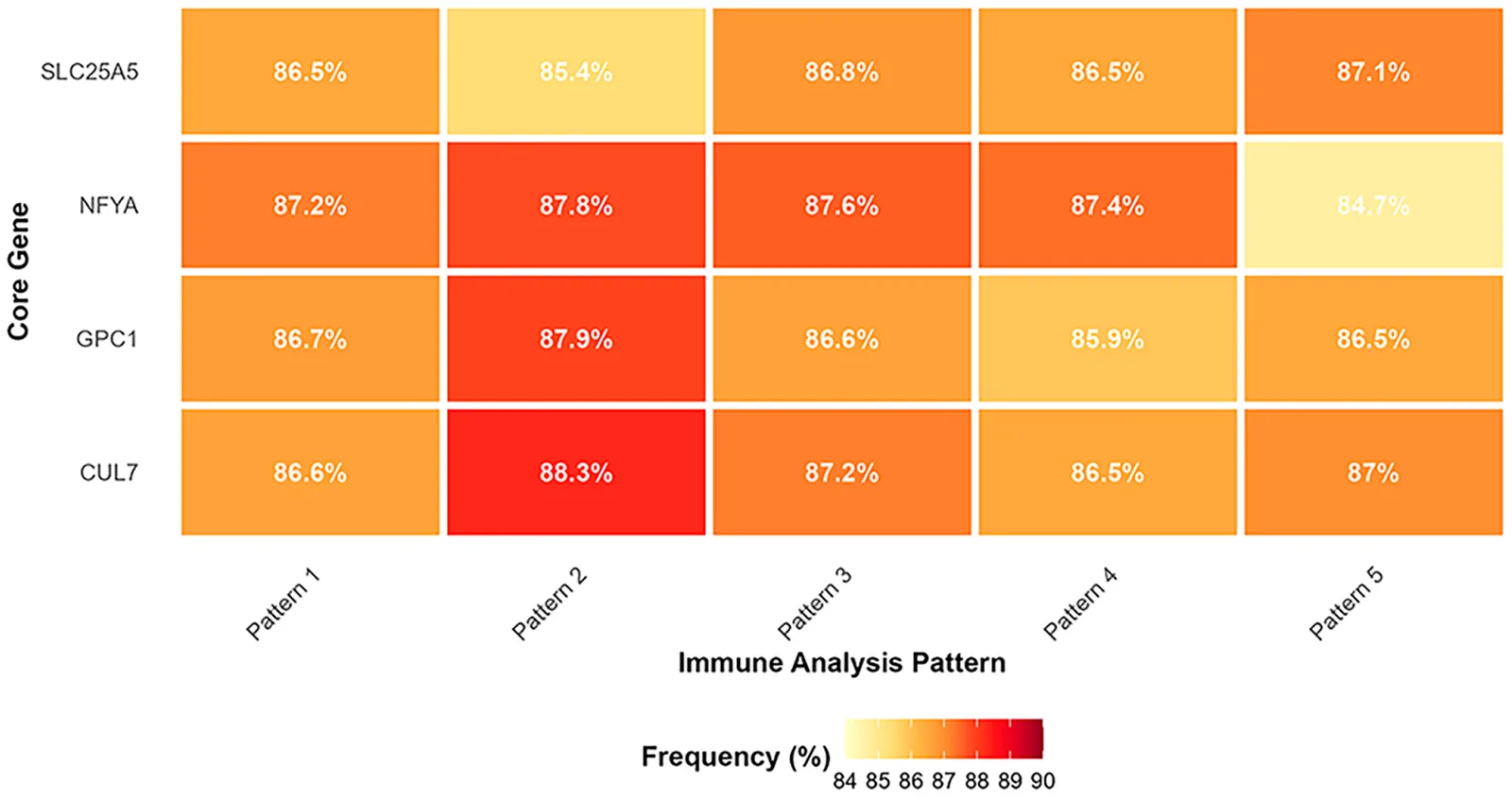
Performance of core genes across distinct immune patterns.

The heatmap (Figure 11) reflects context-dependent core-gene frequency shifts across five immune subtypes. CUL7 and GPC1 peaked in Pattern 2, while NFYA dominated Pattern 2 yet fell to 84.7% in complex Pattern 5, where SLC25A5 rose to 87.1%. CUL7 stayed above 86.5% in most patterns and possessed the best overall stability.

### 3.7. Validation Results of Immunological Analysis Based on GEO Dataset

#### 3.7.1. Verification of Sample Expression Profile Differences

PCA on the GSE39582 dataset distinguished CRC tumor and normal samples (Figure S13). Samples formed largely separated clusters with partial overlap, confirming genome-wide transcriptional divergence between neoplastic and non-tumor colorectal tissues.

#### 3.7.2. DEG Identification Results

**Figure 12:**
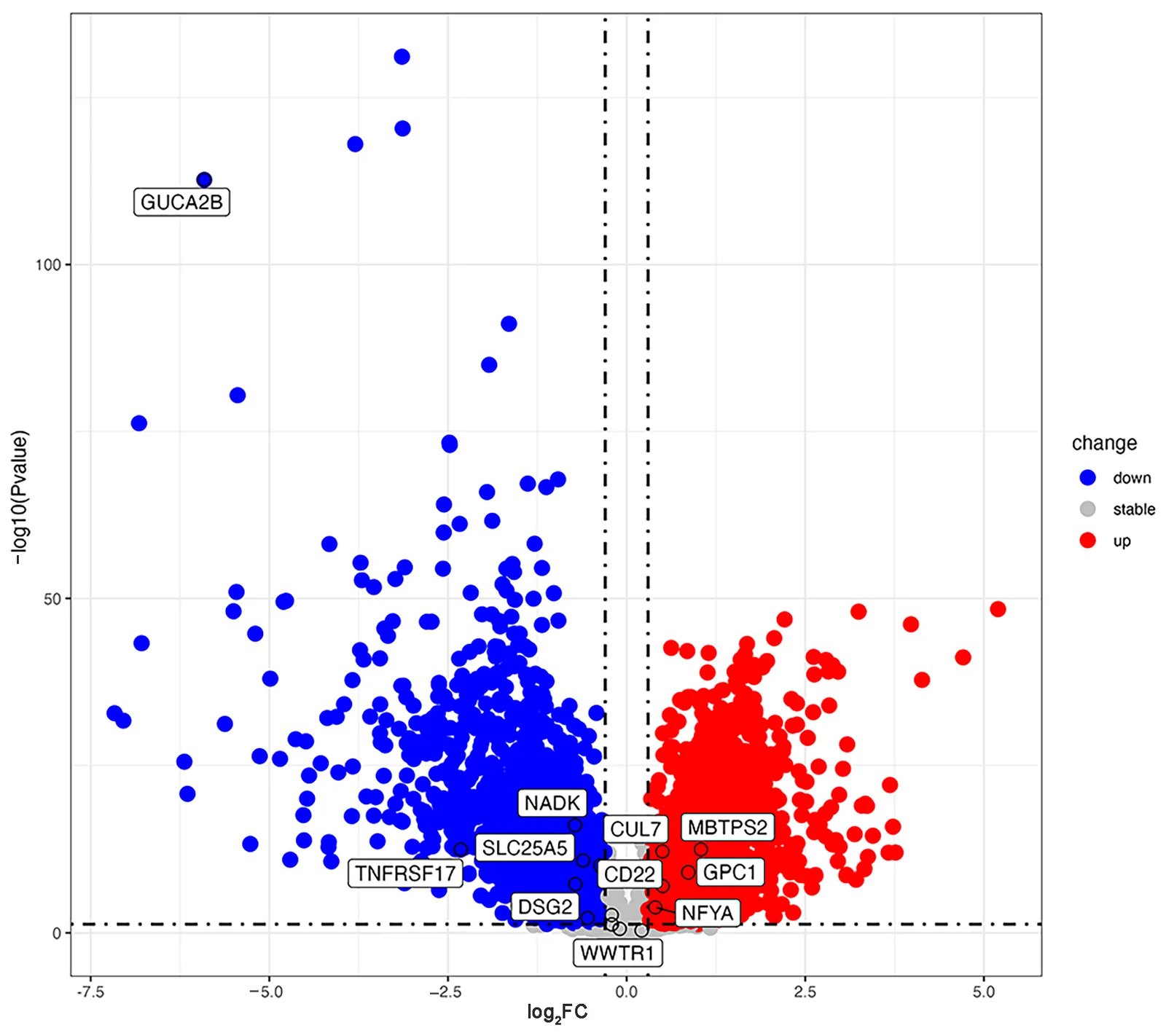
Volcano plot of DEGs between tumor and normal tissues in the GSE39582 cohort.

In total, 7502 DEGs (4593 upregulated, 2909 downregulated) were screened, reflecting widespread transcriptional activation and repression in CRC. The volcano plot (Figure 12) confirmed differential expression of four core genes and multiple auxiliary genes. Notably, the auxiliary gene *GUCA2B* was significantly downregulated, consistent with its high frequency in the 16-head model and supporting its synergistic regulatory role. These results independently validate the biological relevance of core genes identified via the GAT-based screening framework.

#### 3.7.3. Enrichment Analysis Results of DEGs Based on GEO Dataset

**Figure 13:**
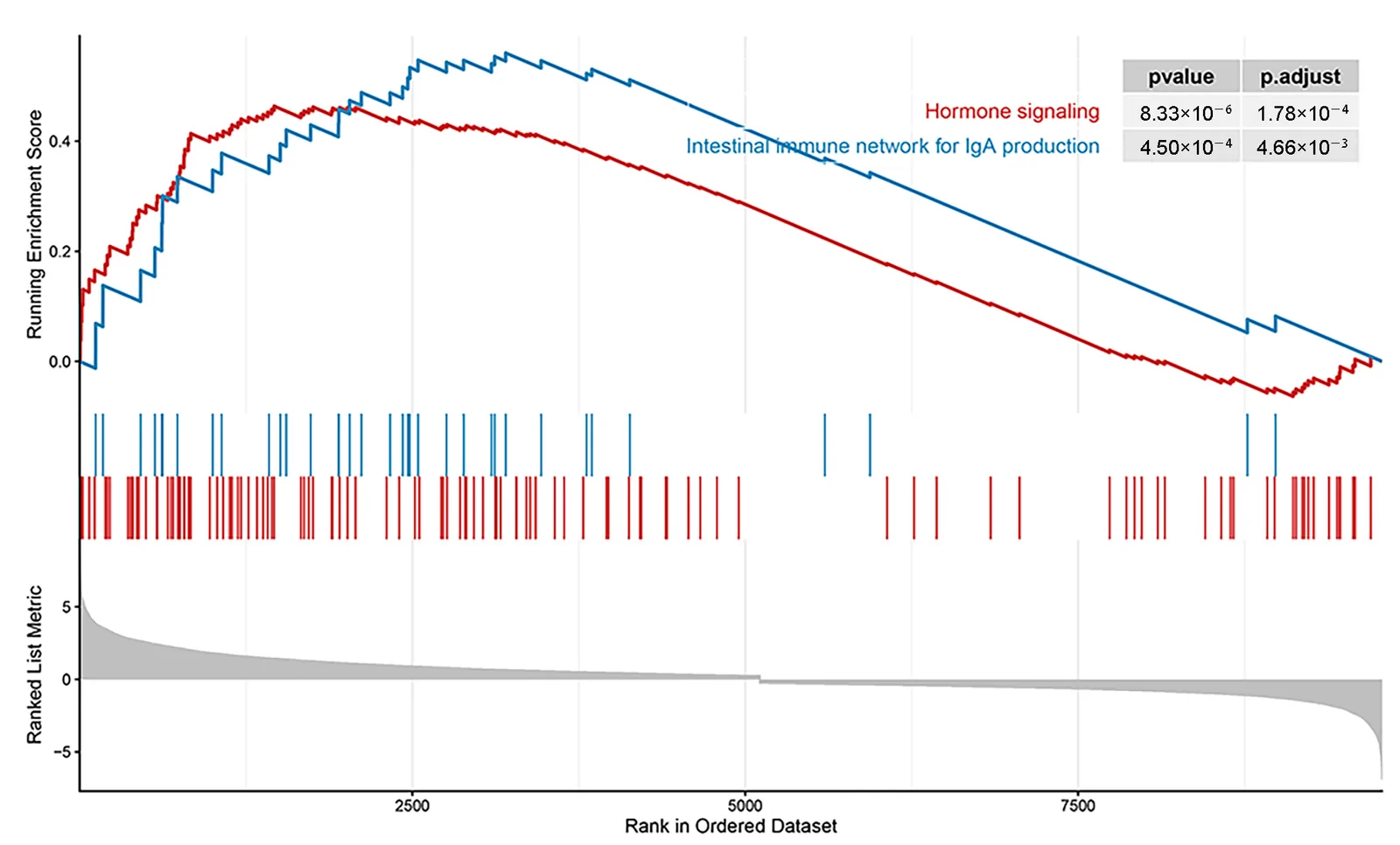
GSEA of core and auxiliary genes based on the GSE39582 dataset.

GSEA of pooled DEGs highlighted tumor- and immune-associated pathways. Two pathways were tightly linked to the core and auxiliary gene module: hormone signaling and intestinal IgA-producing immune network (Figure 13). The hormone pathway modulates CRC cell proliferation and malignant transformation, whereas the IgA network shapes tumor microenvironment (TME) immune infiltration. These data elucidate plausible mechanistic routes by which the core and auxiliary gene set drives CRC progression.

#### 3.7.4. Comparative Immune Microenvironment Associations Between Tumor and Normal Tissues

**Figure 14:**
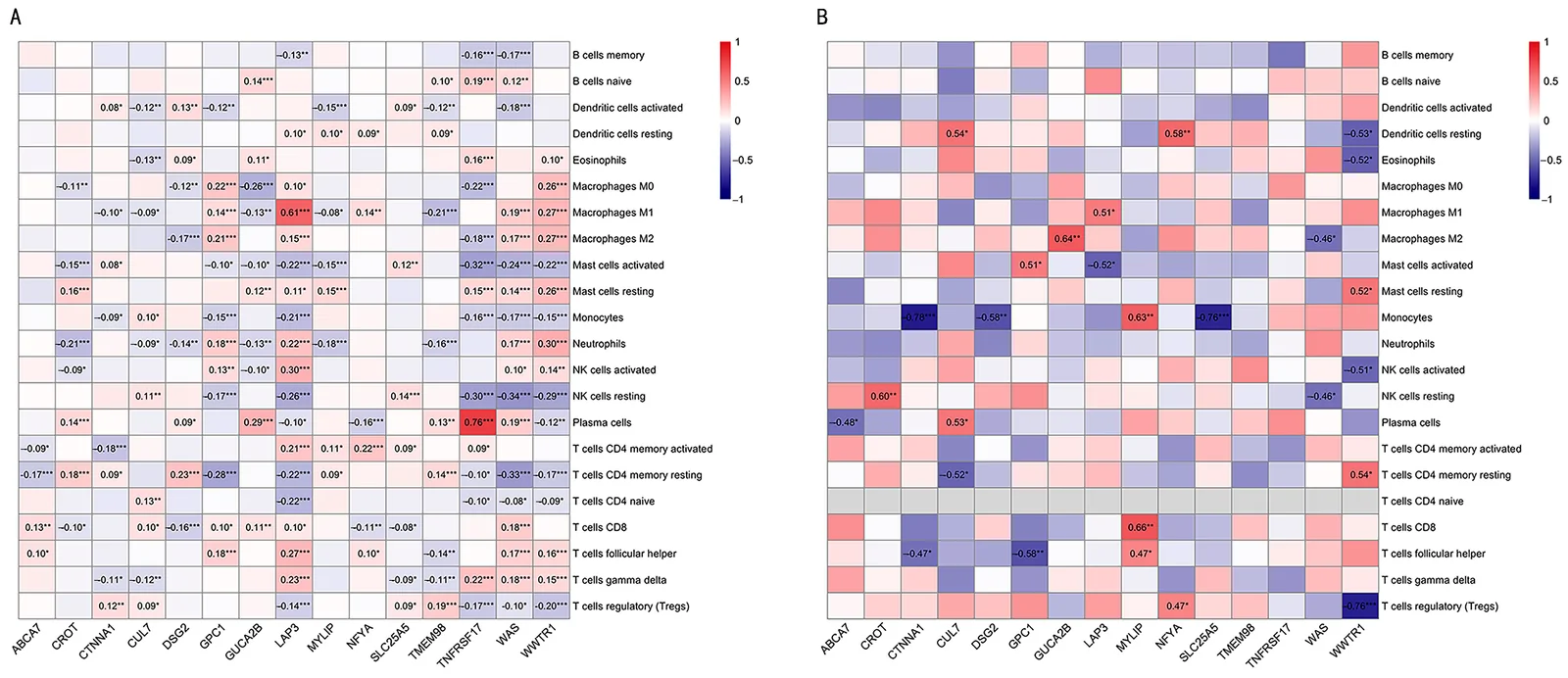
Correlation of core/auxiliary genes with immune cell infiltration in GSE39582. (**A**) Tumor samples. (**B**) Normal samples. Only significant correlations are shown: *** *p* < 0.001, ** *p* < 0.01, * *p* < 0.05.

Correlation analysis based on 566 tumor samples and 19 normal mucosal samples from the GSE39582 revealed markedly distinct gene–immune infiltration regulatory networks (Figure 14A,B). Tumors contained far more numerous and stronger gene–immune correlations (e.g., *TNFRSF17*–plasma cell, *r* = 0.76). Normal mucosa featured sparse weak associations (e.g., *GUCA2B*–M2 macrophage, *r* = 0.64) without robust correlations. Tumor-derived correlations exhibited consistent positive/negative directional trends, whereas normal counterparts were scattered and irregular, indicative of tumor-specific immune network dysregulation. CD4 naive T cells were undetectable in normal specimens, further highlighting intrinsic immune composition disparities.

Many gene immune links were newly acquired, strengthened or polarity reversed in CRC. *GPC1* gained positive correlations with all macrophage subsets exclusively in tumors, supporting its role in tumor associated macrophage reprogramming. The tumor specific positive correlation between *NFYA* and activated CD4 memory T cells suggests its involvement in aberrant T cell responses within TME. The correlation between *TNFRSF17* and plasma cells rose sharply from 0.43 (not statistically significant) in normal tissue to 0.76 in tumor tissue, marking a tumor specific immune signature. Coupling between *LAP3* and M1 macrophages was also enhanced in malignant lesions.

Several polarity shifted interactions were observed: *GUCA2B* shifted from a positive correlation with M0 macrophages in normal tissue to a negative correlation in tumor tissue. The inhibitory association between *WAS* and resting NK cells was strengthened in CRC. *CUL7* established a novel correlation with naive CD4 T cells that existed only in tumor samples.

Four core genes exhibited distinct rewiring of immune associated functions within the TME. *GPC1* underwent prominent functional reprogramming: universal positive correlations with all macrophage populations in tumor tissue contrasted with its negative regulatory relationships with macrophages in normal tissue, a shift that promotes CRC progression. *NFYA* formed tumor exclusive linkages with activated CD4 memory T cells and gained the capacity to regulate T cell activity. *CUL7* only formed sparse weak immune correlations across both tissue types and lacks dominant immunomodulatory effects. *SLC25A5* lost its strong normal exclusive negative correlation with monocytes and displayed broadly weakened immune coupling following TME remodeling.

CRC tissue possesses denser, stronger and more structured gene immune regulatory networks compared with healthy colon tissue. The four core genes experience divergent functional transformations: *GPC1* and *NFYA* acquire tumor specific immune regulatory properties, *SLC25A5* loses its original immune correlations, and *CUL7* exerts minimal effects on the modulation of immune infiltration.

### 3.8. Protein Network

#### 3.8.1. Overall Characteristics of Protein Interaction Networks

**Figure 15:**
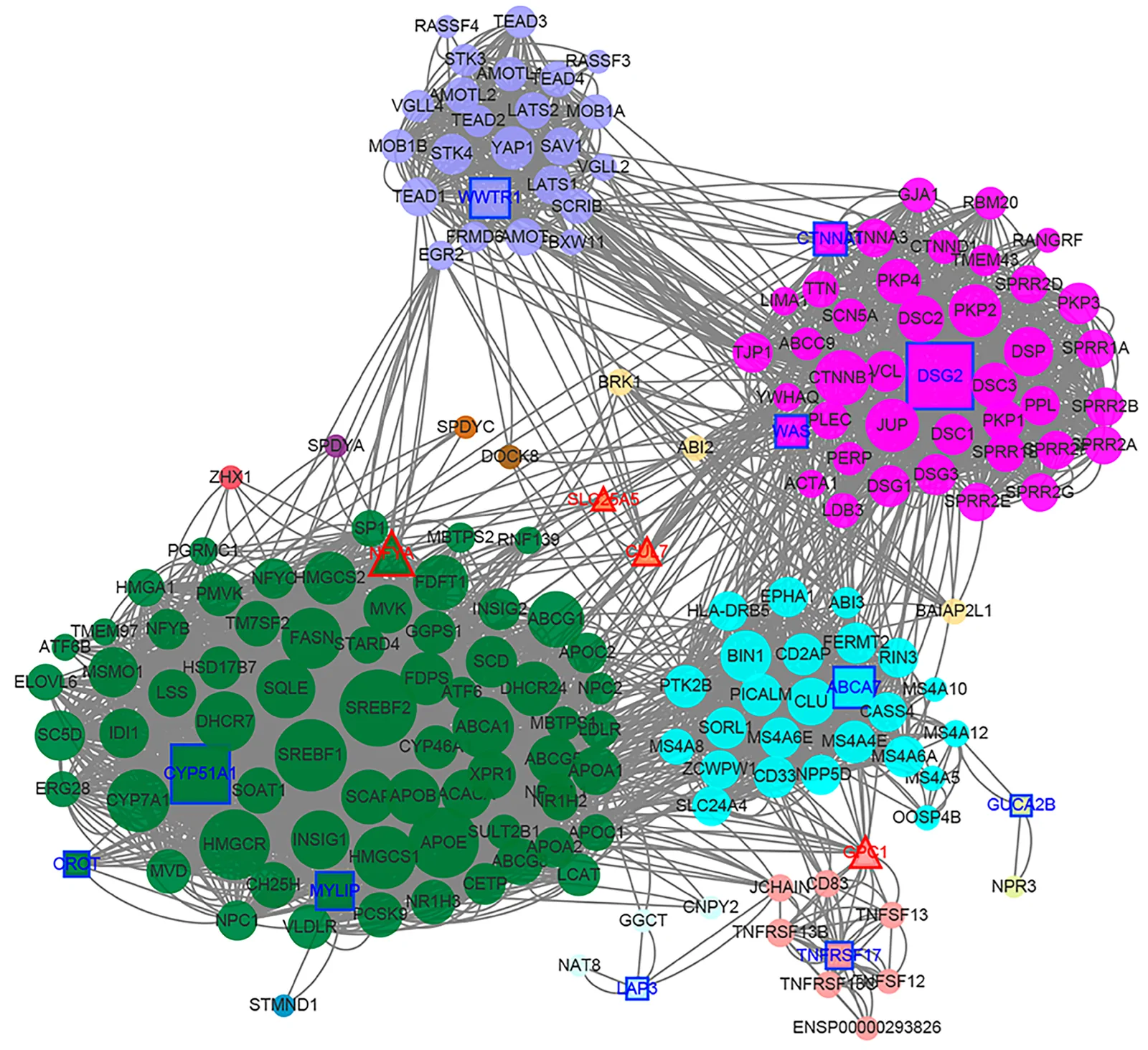
PPI network of proteins encoded by core and auxiliary genes. Core genes are marked as red triangles; auxiliary genes as blue rectangles. Nodes within the same subnetwork share identical color coding.

Leiden clustering partitioned the STRING-based PPI network into four tightly interconnected functional modules (Figure 15). The protein products translated from the four core genes (*CUL7*, *GPC1*, *NFYA*, *SLC25A5*) occupied pivotal bridging positions within the network. The proteins of *CUL7* and *SLC25A5* served as central hubs that connected separate subnetworks. Although these core genes exhibited only mild differential expression, their encoded proteins dominated the topological structure of the interactome. By contrast, proteins derived from auxiliary genes were dispersed across all clusters to assemble a unified regulatory interactome.

#### 3.8.2. Enrichment Analysis of Subnetworks

The dark green module was associated with cholesterol and lipid metabolism. GO enrichment highlighted cholesterol biosynthesis, sterol transport and lipid homeostasis (Figure S14), with KEGG results pointing to AMPK, PPAR and atherosclerosis pathways (Figure S15). This module remodels tumor lipid metabolism to sustain energy supply and membrane synthesis, and potentially reshapes the TME via steroid hormone signaling to drive CRC development. Next, the light purple module corresponded to Wnt and Hippo signaling. This cluster was enriched in canonical Wnt and Hippo pathways governing organ development and oncogenic proliferation, accompanied by GO terms related to organ and epidermal growth (Figures S16 and S17). Conserved Hippo signaling functionally connects core genes to developmental pathways underlying CRC initiation and progression. The purple red module featured cell junction and electrophysiology functions, with enrichment centered on cell to cell junction assembly and ion-channel-related electrophysiological processes (Figures S18 and S19). Dysregulated junction proteins facilitate EMT and metastatic progression, offering a mechanistic clue for core genes to modulate CRC metastasis. Finally, the cyan module covered cytoskeleton organization and immune phagocytosis. Genes within this module regulate actin cytoskeleton rearrangement and cell motility, and converge on FcγR-dependent phagocytosis according to KEGG analysis (Figures S20 and S21). Consistent with earlier findings regarding IgA and B cells, this module mediates antitumor immunity by adjusting cytoskeleton dynamics in phagocytes.

Core and auxiliary genes cooperatively govern four biological axes: lipid metabolism, developmental signaling, intercellular adhesion and immune cytoskeleton. Instead of functioning in an isolated pathway, the core module integrates metabolism, signal transduction, cell communication and immunity to construct CRC TME. Notably, the coexistence of lipid and phagocytosis modules implies critical metabolic–immune crosstalk during tumor progression.

### 3.9. Results Related to TF-Associated Regulatory Networks

**Figure 16:**
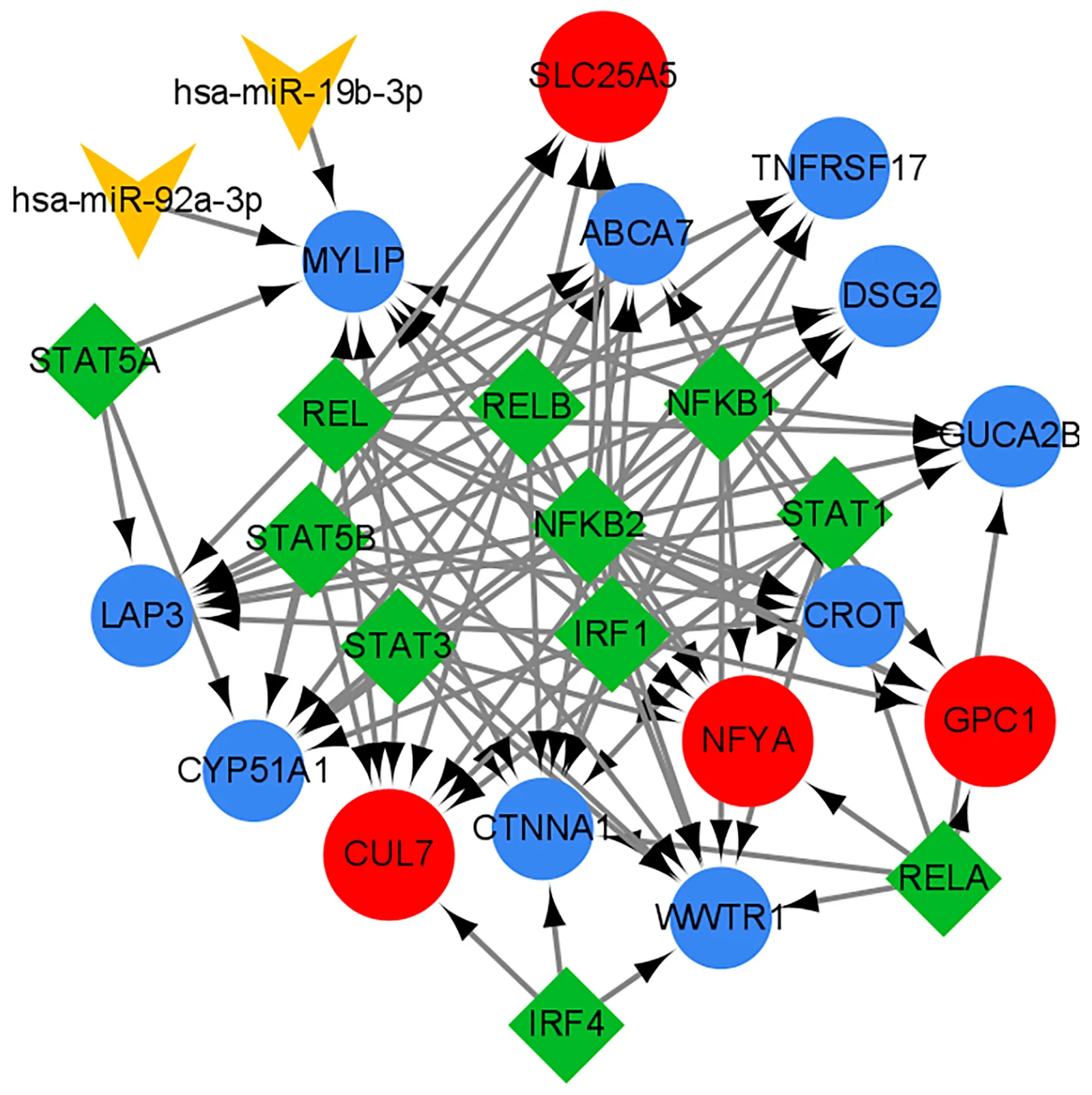
Regulatory network comprising core/auxiliary genes, transcription factors and miRNAs. Red corresponds to core genes, blue to auxiliary genes, green to transcriptional regulators, and yellow to miRNAs.

A regulatory network containing 109 interactions was built with 15 core/auxiliary genes, 11 TFs and 2 miRNAs (Figure 16), hierarchically dominated by NF-κB, STAT and IRF immune-related transcription factors.

NF-κB members (REL, NFKB1, NFKB2, RELB, RELA) regulated 80–93% of target genes. REL was the top hub controlling 14 genes (93%) and linking inflammatory cues to downstream targets. STAT1/3/5A/5B formed the secondary regulatory layer responsive to cytokines. IRF1 broadly modulated 13 genes (87%), whereas IRF4 specifically regulated only three genes. Overall, the core module is predominantly governed by immune/inflammatory TFs, especially NF-κB, coordinating immune infiltration and CRC progression.

As the sole target controlled by nearly all TFs (except STAT5A), NFYA acts as a central network hub. Being a component of basal transcription complex, NFYA integrates upstream immune signals to remodel global transcription in CRC cells.

Immune TFs widely target metabolic auxiliary genes such as *CYP51A1*, *ABCA7* and *MYLIP*, all co-regulated by nine TFs. The oncogenic miR-17-92-derived miRNAs (hsa-miR-19b-3p, hsa-miR-92a-3p) further inhibit *MYLIP* post-transcriptionally, forming a feedback loop: NF-κB drives *MYLIP* transcription to restrain cholesterol intake, while the two miRNAs counteract this inhibition. Disruption of this immune–metabolic balance disturbs cholesterol homeostasis and accelerates CRC progression.

NF-κB/STAT/IRF TFs cooperatively control core *NFYA* and pivotal metabolic genes, with two miRNAs adding post-transcriptional regulation. This cascade bridges immune activation and metabolic reprogramming in TME.

### 3.10. Single-Cell Analysis

**Figure 17:**
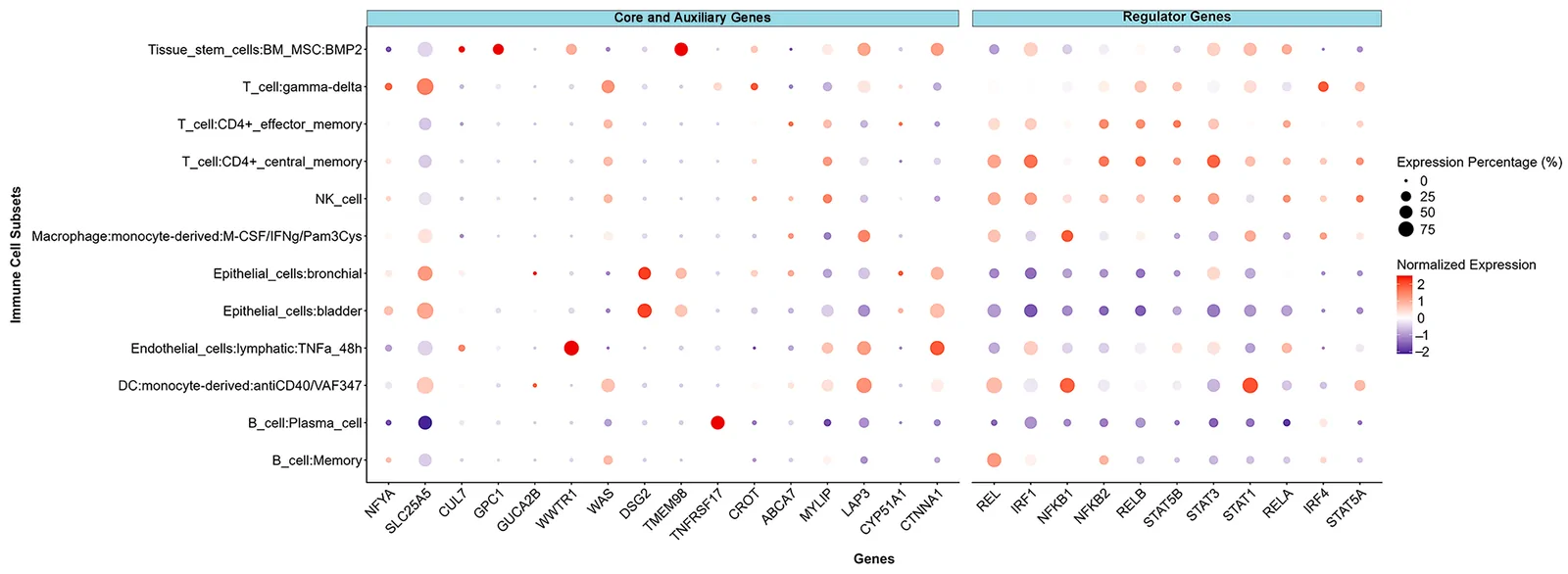
Single-cell expression profiles of core genes, auxiliary genes and transcriptional regulators across tumor immune microenvironment cell subpopulations.

Single-cell transcriptome analysis on GSE132257 was processed via Seurat for normalization, clustering and t-SNE, and annotated by SingleR (Figure 17). Core genes *NFYA, CUL7, GPC1* were broadly lowly expressed across all cell subsets, whereas mitochondrial transporter gene *SLC25A5* maintained universal high expression like a housekeeping gene.

Auxiliary genes featured prominent cell-type specificity. Epithelial cells highly expressed *DSG2*, *TMEM98*, *CTNNA1*. Lymphocytes and myeloid immune cells enriched *WAS*, *MYLIP*, *LAP3*. *TNFRSF17* specifically marked plasma cells. NF-κB/STAT/IRF transcription factors were moderately or highly enriched in immune cells, especially activated subsets. Mesenchymal and lymphatic endothelial populations uniquely upregulated *GPC1* and *WWTR1*.

Core genes sustain basal cellular homeostasis. Notably, *SLC25A5* serves as a potential internal control. Cell specific auxiliary genes define cellular identity, while immune-related transcription factors govern cell activation and fate decisions. Distinct sets of genes act synergistically to determine cell phenotype and functional status.

### 3.11. Drug Sensitivity Prediction and Molecular Docking Analysis

#### 3.11.1. Drug Sensitivity Prediction

Based on the expression characteristics of core and auxiliary genes, this study successfully predicted the drug sensitivity of CRC samples to multiple agents. The top 10 most frequently occurring candidate drugs were identified: mitoxantrone (antineoplastic antibiotic), oxaliplatin (third-generation platinum-based anticancer drug), obatoclax mesylate (BCL-2 inhibitor), actinomycin D (also known as dactinomycin), sapitinib (EGFR/HER2 inhibitor), JQ1 (BET bromodomain inhibitor), luminespib (HSP90 inhibitor), KU-55933 (ATM kinase inhibitor), PF-4708671 (S6K1 inhibitor), and paclitaxel (classic anticancer drug).

#### 3.11.2. Molecular Docking Results

Molecular docking was performed using protein products encoded by core and auxiliary genes to screen potential targeted drugs. Multiple candidate compounds including oxaliplatin, sapitinib, and PF-4708671 were subjected to molecular docking. Table S3 shows all docking results. Table S4 lists the top 10 docking results between candidate drugs and metabolism-related target genes. Their binding energies ranged from −8.29 to −7.79 kcal/mol. Table S5 presents the optimal binding partners for the four core genes. Each core gene matched one preferred compound, with binding energies spanning −7.78 to −6.31 kcal/mol.

### 3.12. Functional Perturbation Characteristics of GPC1 and SLC25A5 in HT29 Cells

**Figure 18:**
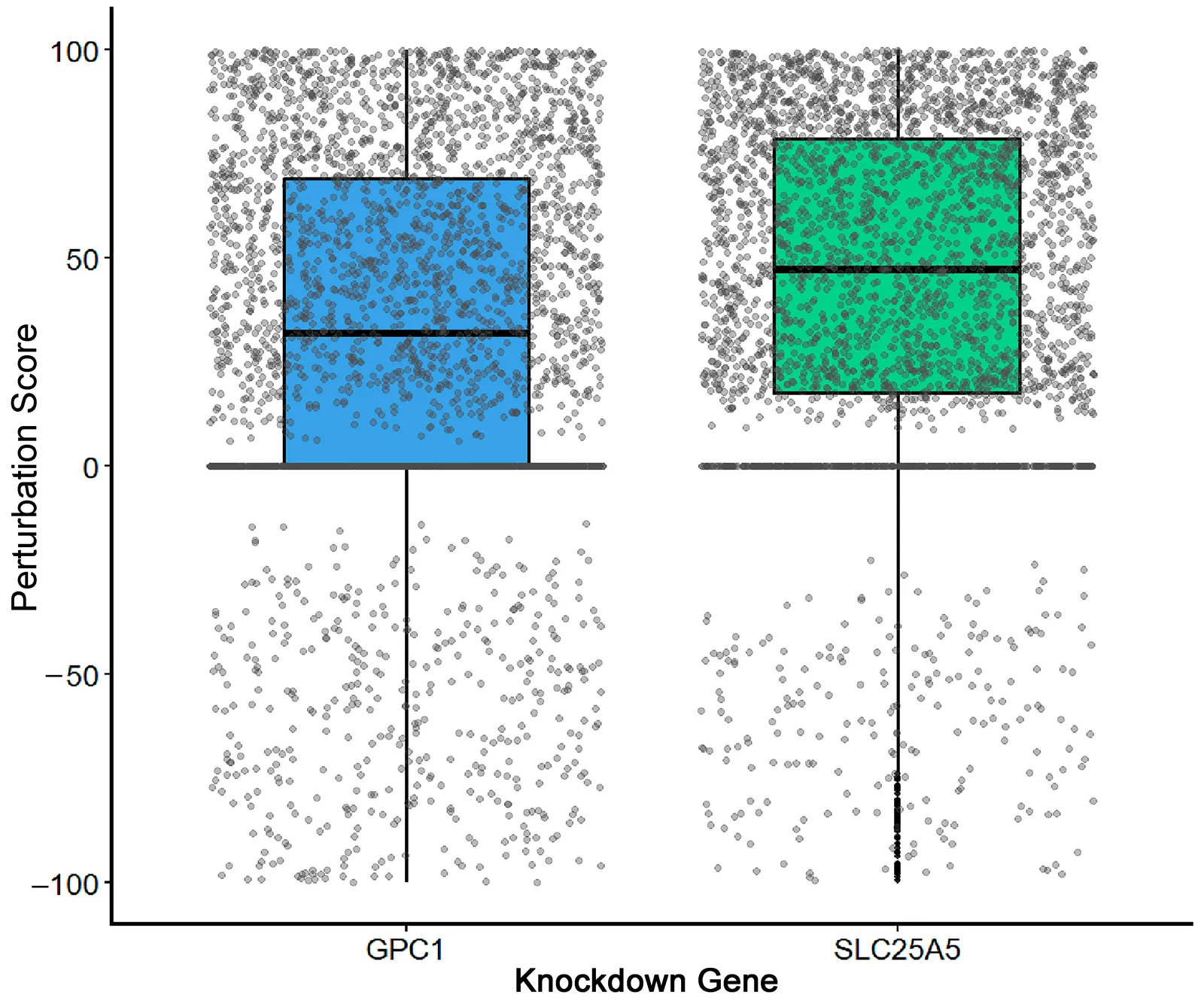
In silico perturbation of core genes *GPC1* and *SLC25A5*.

LINCS L1000 knockdown data were used to compare transcriptome perturbations of *GPC1* and *SLC25A5* in HT29 cells via box plots (Figure 18). *GPC1* knockdown produced a median perturbation score of ~30 and globally elevated most downstream transcripts. By contrast, *SLC25A5* silencing achieved a higher median (~50) with wider score dispersion, driving more intense and heterogeneous transcriptional remodeling.

Both genes triggered profound genome-wide expression shifts, verifying their essential regulatory functions instead of random prognostic markers. The predominance of positive perturbation values indicates their intrinsic transcriptional repression. Gene knockdown alleviates such inhibition and activates downstream cascades, with *SLC25A5* possessing stronger global regulatory potency.

## 4. Discussion

This study established a rigorous multi-omics analytical pipeline to characterize the epigenetic and immune regulatory landscapes of CRC. The main objectives were to identify reliable prognostic immune regulatory genes, and to promote epigenetic subtyping and precision therapy for CRC.

We first integrated three multi-omics datasets from the TCGA database. A gene functional similarity network was then constructed based on KEGG and Reactome pathway annotations combined with the PFC index. By intersecting UniProt-annotated proteins encoded by histone modification-related genes with DEGs from TCGA, we obtained a panel of high-confidence tumor-specific epigenetic regulatory candidate genes. We applied a two-layer GAT to generate biologically interpretable gene embeddings. We further screened core prognostic genes using RSF and LightGBM algorithms. Their biological roles were interpreted via SHAP analysis, and prognostic performance was validated using log-rank tests. The GSE39582 cohort was used for external validation across independent datasets. Subsequently, we performed STRING-based PPI network analysis and functional enrichment analysis to explore the core biological pathways of these candidate genes. TF and miRNA regulatory network analysis, together with single-cell RNA-sequencing analysis, further elucidated the upstream regulatory mechanisms and cell-type distribution of core genes within the TIME. Lastly, we conducted drug screening and molecular docking assays to evaluate compound-protein binding affinity. In silico perturbation assays revealed that dysregulated expression of *GPC1* and *SLC25A5* triggers extensive transcriptomic reprogramming, which supports their essential regulatory functions in CRC progression. Our results provide solid preliminary evidence for the preclinical translational potential of the identified genes.

In this study, we built the initial HMRG candidate pool solely by extracting all proteins harboring histone modification-recognition domains from UniProtKB. No additional manual curation was performed to separate proteins that specifically recognize histone marks from other domain-carrying proteins that only interact with cytoplasmic non-histone substrates. This simplified retrieval workflow may retain a small proportion of proteins without authentic histone-binding capacity.

Nevertheless, the core aim of our research is consistent with the manuscript title: we do not intend to systematically characterize biochemically verified, strictly canonical histone modification readers, but rather to screen domain-bearing candidate histone mark binders that participate in CRC immune microenvironment remodeling.

To mitigate the inherent limitations of our preliminary broad screening, we adopted a multi-step omics and functional validation framework, including differential expression analysis, GAT-based multi-omics integration, prognostic machine learning modeling, immune infiltration correlation analysis, single-cell transcriptome profiling, molecular docking simulation, and in silico gene perturbation assays. Only genes supported by consistent evidence of aberrant tumor expression, prognostic predictive power, and immune regulatory activity were retained as core and auxiliary functional HMRGs for subsequent in-depth exploration. Even though our initial candidate collection was broad, multi-dimensional phenotypic evidence confirms that the final gene set is biologically relevant to CRC progression and immune remodeling. In follow-up studies, we will add manual annotation filtering and biochemical experiments to more rigorously screen histone-specific reader proteins.

Given the moderate expression changes in immune-related genes in CRC, we customized the differential gene screening threshold as |log_2_FC| > 0.3 and adj.*p*-value < 0.05 [57,58]. After obtaining preliminary differentially expressed immune-related genes with these criteria, we abandoned reliance on arbitrary statistical cutoffs and constructed a progressive three-tier immune assessment system. This multi-layer framework, which prioritizes biological function and clinical relevance, covers clinical prognosis, molecular mechanisms and TIME characteristics. The first tier evaluated immunotherapy responsiveness to prioritize genes with direct translational potential [59,60]. The second tier combined immune checkpoint expression and immune cell abundance analysis to uncover the molecular mechanisms of tumor immune regulation [61,62]. The third tier comprehensively profiled immune phenotypes, immune escape patterns and spatial microenvironmental traits for a full assessment of the TIME [63,64].

We applied this hierarchical framework to define five distinct immune patterns and screen core and auxiliary genes. Redundant immune parameters were excluded to avoid information redundancy, as core immune signaling pathways were fully captured within this six-dimensional evaluation system [65]. Genes showing robust regulatory effects across all three tiers were prioritized for follow-up analysis. Ultimately, four genes (*CUL7*, *GPC1*, *NFYA* and *SLC25A5*) which exerted prominent regulatory effects across all three tiers were determined as the key immune regulators in CRC.

The biological credibility and robustness of our core–auxiliary gene module were fully validated by the existing literature. Consistent with published CRC research, the four core genes exert irreplaceable regulatory functions in tumor progression and immune remodeling. CUL7 drives CRC malignant phenotypes by modulating p53 and Wnt/β-catenin signaling and serves as a biomarker for poor prognosis [66]. GPC1 facilitates CRC proliferation and migration via activating TGF-β signaling and is tightly correlated with tumor macrophage infiltration [67]. NFYA suppresses ferroptosis to promote CRC progression and is closely associated with activated memory CD4^+^ T cell infiltration [68]. In contrast, SLC25A5 plays a tumor-suppressive role by inhibiting the MAPK pathway and maintaining cellular metabolic homeostasis [69]. For auxiliary genes, low-frequency *GUCA2B* is post-transcriptionally regulated by specific miRNAs to modulate CRC cell proliferation and migration [70]. WWTR1 is a key regulator of oxaliplatin sensitivity, and our molecular docking results confirmed its high-affinity binding with PF-4708671 (ΔG = −7.71 kcal/mol), verifying its druggable potential in CRC targeted therapy [71]. Downregulated *DSG2* correlates with distant metastasis and adverse clinical outcomes, acting as an independent prognostic biomarker for CRC [72]. These literature findings corroborate the biological authenticity of our screened gene module.

Numerous independent studies have verified that the core and auxiliary genes identified in this work are closely associated with the initiation and progression of CRC, providing literature evidence for the biological authenticity of this gene module. Even so, the present study has notable limitations in biological mechanism validation and clinical translation. All conclusions of this research are derived from bioinformatic computational analyses, external transcriptomic cohort validation and in silico perturbation assays, without supporting wet-lab experiments such as cellular and animal studies. Although multi-layer bioinformatic analyses improve the reliability of this gene signature, the absence of tangible experimental evidence restricts the direct clinical application of these biomarkers. Collectively, the theoretical mechanism we elucidated via systematic multi-omics analyses—where core genes participate in tumor immune regulation to enable patient risk stratification—still offers valuable insights for prognostic stratification and individualized precision therapy of CRC.

A multi-scale validation pipeline combining multi-omics datasets, the independent GSE39582 cohort and single-cell transcriptomic data effectively reduced bias from individual datasets and verified the robustness of the identified gene module.

Differential expression analysis based on the GSE39582 transcriptome dataset confirmed that four core genes and multiple auxiliary genes exhibit significant expression abnormalities in CRC. GSEA enrichment analysis results indicated that these genes are predominantly enriched in hormone signaling pathways and immune regulatory networks associated with intestinal IgA synthesis, playing a role in maintaining immune homeostasis. Immunological association analysis revealed that, compared to the sparse, weak, and irregular gene–immune association patterns observed in normal mucosa, the immune regulatory networks in CRC tissues are more dense, exhibit stronger interaction intensities, and demonstrate stable correlation directions. The tumor microenvironment undergoes extensive reprogramming, with core genes mediating numerous newly established or significantly enhanced immune interactions.

Single-cell transcriptome data further confirm that NFYA, CUL7, and GPC1 exhibit broad-spectrum low expression across various cell subpopulations, whereas SLC25A5 demonstrates stable high expression in all cells. Auxiliary genes demonstrate distinct cell-type-specific expression profiles, with each type of epithelial, immune, mesenchymal, and endothelial cell possessing characteristic marker genes. Core genes primarily participate in maintaining cellular homeostasis, while cell-specific auxiliary genes, together with immune-related transcription factors, collectively regulate cellular lineage identity, activation levels, and biological functional phenotypes. These findings unequivocally demonstrate that the core and auxiliary genes identified through multi-omics screening play pivotal regulatory roles in the remodeling of the tumor immunomicroenvironment in CRC.

We adopted the GAT to address the drawbacks of traditional machine learning methods in biological network mining. By embedding gene functional similarity networks, the GAT model accurately reconstructs complex nonlinear gene regulatory interactions, which aligns well with the intrinsic properties of epigenetic modulation. Equipped with a 16-head dynamic attention mechanism, this architecture enables efficient capture of heterogeneous gene–gene crosstalk, filters technical noise, and pinpoints biologically relevant synergistic gene pairs. Notably, the core genes prioritized by GAT exhibited strong consistency across external cohort validation and single-cell transcriptomic profiling. As a reliable framework tailored for high-dimensional sparse biological networks, GAT facilitates the interpretable screening of pivotal epigenetic and immunoregulatory genes and significantly elevates the reliability and predictive performance of our gene identification pipeline.

The two-layer GAT structure was chosen to balance feature learning capacity, training stability and biological interpretability, while avoiding the over-smoothing issue commonly seen in deep graph neural networks. Although deeper layers can capture more complex topological patterns, they tend to produce homogenized gene embeddings, weaken the functional specificity of individual genes, raise computational costs and increase the risk of overfitting, which ultimately reduces the interpretability of outputs. Our optimized two-layer design delivers favorable analytical performance. The first layer extracts multi-omics topological features of genes, and the second layer refines feature representation through batch normalization to stabilize model training. This architecture effectively learns nonlinear regulatory interactions among histone modification-related genes. Meanwhile, it preserves the distinct functional characteristics of each gene, making it well suited for multi-omics data integration and the reliable identification of core epigenetic and immune regulators in CRC.

Notably, this study prioritizes the exploration of biological mechanisms and clinical relevance rather than pursuing extreme model prediction accuracy. As reflected in our results, the C-index of the GAT model drops sharply from 0.906 in the TCGA training set to 0.653 in the external GSE39582 test cohort, which clearly reveals mild overfitting of the 16-head GAT architecture. Such overfitting is a common trade-off in high-dimensional biological network analysis, largely driven by dataset-specific noise captured by multi-head attention layers and the limited sample size of matched multi-omics cohorts. Although the GAT model carries inherent overfitting risks and weakened generalization on unseen samples, multi-level independent validations based on TCGA multi-omics data, external GEO cohorts, and single-cell transcriptomic data fully confirm the biological functionality of the identified gene modules and offset the adverse impact of model overfitting. In addition, all functional and phenotypic validations in this study are independent of model fitting performance, further eliminating potential analytical bias caused by overfitting. We also objectively reported the model’s limitations throughout the manuscript to guarantee the rigor and reproducibility of our research findings.

**Figure 19:**
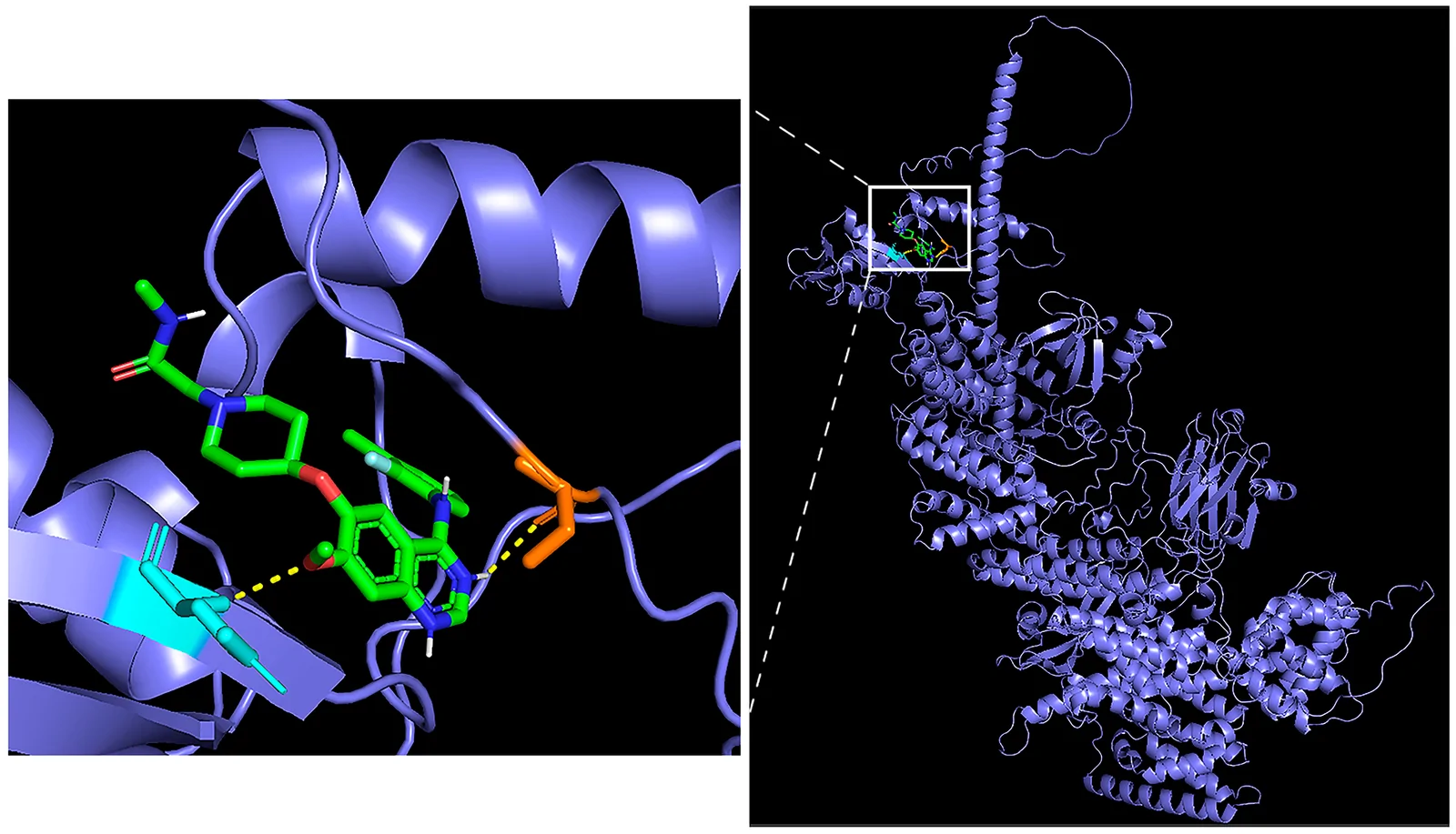
Molecular docking of sapitinib with CUL7. Glutamine (GLN, blue-green) at position 192 and phenylalanine (PHE, orange) at position 194 of the CUL7 protein.

**Figure 20:**
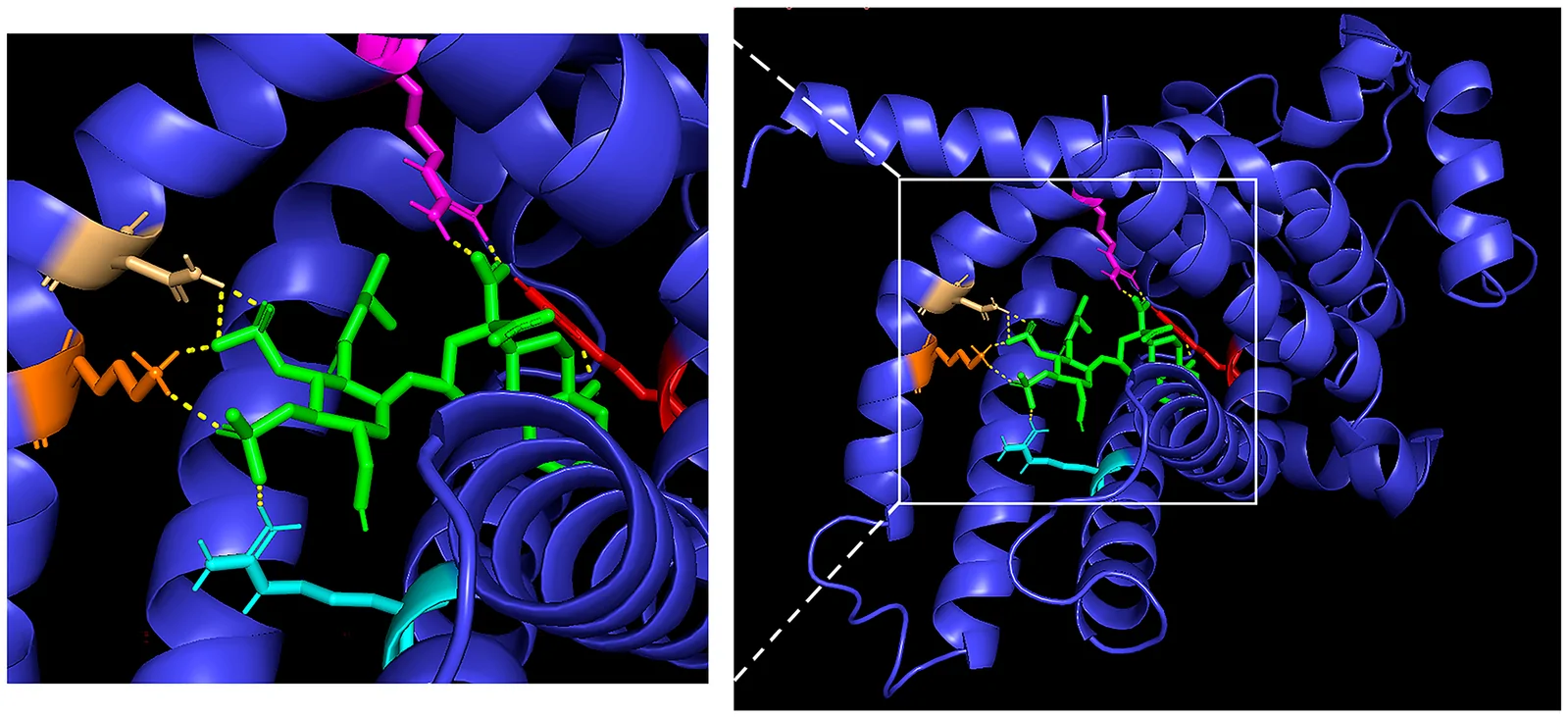
Molecular docking of oxaliplatin with SLC25A5. Arginine (ARG, magenta) at position 256, asparagine (ASN, cyan) at position 260, isoleucine (ILE, orange) at position 198, lysine (LYS, red) at position 173, and tyrosine (TYR, light orange) at position 173 of the SLC25A5 protein.

**Figure 21:**
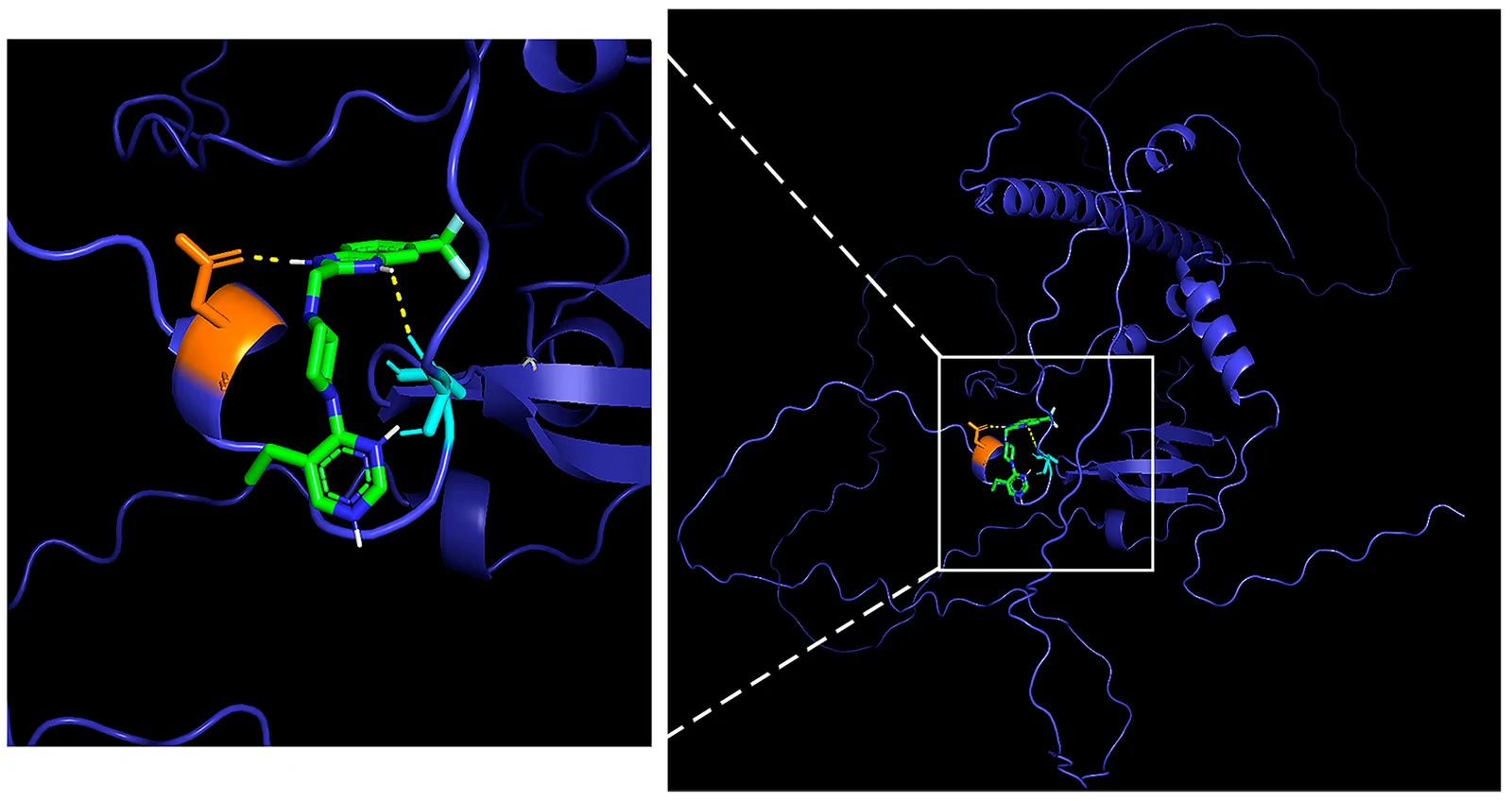
Molecular docking of PF-4708671 with WWTR1. Aspartate (ASP, orange) at position 362 and threonine (THR, blue-green) at position 367 of the WWTR1 protein.

Molecular docking analysis was performed to screen potential therapeutic compounds and evaluate their druggability for CRC precision treatment. As a core oncogenic driver regulating p53 and Wnt/β-catenin signaling, CUL7 showed strong binding affinity with Sapitinib (ΔG = −7.78 kcal/mol). Structural analysis (Figure 19) verified stable hydrogen bond interactions at the GLN192 and PHE194 residues of CUL7 protein, suggesting that this EGFR inhibitor exerts anti-tumor effects by targeting CUL7 to block oncogenic signaling. The mitochondrial metabolism-related protein SLC25A5 exhibited high binding affinity with the first-line chemotherapeutic agent oxaliplatin (ΔG = −7.48 kcal/mol), with binding interactions occurring at ARG256, ASN260, ILE198, LYS173, and TYR173 residues (Figure 20). This finding expands the canonical DNA-damaging mechanism of oxaliplatin, revealing a novel mitochondrial-targeting mode to overcome CRC chemoresistance. Beyond targeting *CUL7*, Sapitinib also binds tightly to metabolic auxiliary genes *MYLIP* (ΔG = −8.14 kcal/mol) and *LAP3* (ΔG = −8.11 kcal/mol), enabling simultaneous inhibition of lipid and amino acid metabolic reprogramming to enhance anti-tumor efficacy and reduce drug resistance. PF-4708671 displayed robust binding affinity with the Hippo pathway effector WWTR1 (ΔG = −7.71 kcal/mol) via interactions with ASP362 and THR367 residues (Figure 21), indicating its potential to suppress CRC stemness and metastasis. Its ultra-high affinity for CYP51A1 (ΔG = −8.29 kcal/mol) further enables targeted intervention in CRC cholesterol metabolism.

Our molecular docking analysis has several inherent limitations. First, computational docking merely prioritizes potential ligand–protein binding modes and cannot replace biochemical experiments to verify real binding and functional pharmacological effects; all docking-derived conclusions should be interpreted cautiously. Second, we simplified transmembrane proteins as soluble structures without constructing lipid bilayer environments during simulation, which may affect the accuracy of predicted binding poses and binding energies. Third, partial protein domains only possess moderate structural confidence (pLDDT = 70–85) from AlphaFold modeling, which may introduce minor structural uncertainty into subsequent docking calculations. Future research will integrate membrane structural modeling and in vitro binding assays to validate these computational interaction predictions.

Based on molecular docking outcomes, we put forward a tiered precision therapeutic strategy adapted to diverse clinical scenarios of CRC. Sapitinib is recommended as the preferred monotherapy for patients with high CUL7 expression and tumor metabolic disorders. PF-4708671 can be used as an alternative agent for metastatic, stem cell-like and drug-resistant CRC. Moreover, combined regimens of oxaliplatin plus Sapitinib or PF-4708671 produce synergistic anti-tumor effects. These combinations co regulate DNA damage repair, tumor metabolic reprogramming and cancer stemness suppression, and hold great translational potential for the treatment of advanced and refractory CRC.

The candidate drugs identified in this work complement and improve current CRC treatment regimens, helping to address key challenges in precision therapy and drug resistance. Oxaliplatin acts on SLC25A5 via a novel mechanism, extending its classical DNA damage-related function and providing new evidence for treating patients with high SLC25A5 expression. The clinically investigated agent sapitinib targets CUL7, MYLIP and LAP3, which offers a feasible approach to overcome resistance to EGFR inhibitors in CRC. PF-4708671 suppresses cancer stemness by targeting WWTR1, showing distinct advantages for refractory cases.

Although the proteins encoded by these core genes are not conventional targets of mainstream CRC drugs, they exert critical functions in TIME remodeling and immune infiltration. GPC1 modulates macrophage infiltration and polarization, supporting its combined use with TAM-targeted therapy. NFYA regulates responses of activated memory CD4^+^ T cells and may enhance the efficacy of immune checkpoint inhibitors. TNFRSF17 is strongly correlated with plasma cell abundance, implying promising application prospects for combination with B cell-based immunotherapy.

Overall, this study reveals previously unreported epigenetic regulatory patterns and drug response features in CRC. It bridges the gap between fundamental epigenetic research and clinical practice, and provides solid theoretical evidence for optimizing combination regimens and precision treatment strategies for CRC.

This study has several inherent limitations that should be noted. First, all analyses relied on retrospective public datasets, which may cause selection bias and restrict the clinical generalizability of our results. Second, gene function validation and drug targeting evidence were solely derived from in silico analysis. Further in vitro and in vivo experiments are therefore needed for solid verification. Third, the two-layer GAT model is unable to fully capture the highly complex topological interactions within epigenetic regulatory networks. Fourth, this work focused only on histone reader domains, without investigating other types of epigenetic modifications. In addition, the GAT and RSF models show clear overfitting, a key limitation that points to an important direction for future model optimization.

Patient heterogeneity analysis is a core component of modern CRC precision oncology, yet we only partially explored this dimension in the current work. Clinical practice commonly stratifies CRC patients using well-established molecular subgroups, including MSI/MSS status, CMS consensus subtypes, BRAF/KRAS oncogenic mutation profiles and tumor mutational burden. Restricted by incomplete matched multi-omics data annotated with these molecular subtypes in the public cohorts, we could not conduct stratified survival and predictive analyses of our HMRG signature across the above clinical subgroups. Theoretically, our risk scoring system can serve as an auxiliary stratification marker complementary to existing molecular classification frameworks. When combined with MSI status, CMS typing or driver mutation information, this epigenetic signature can further refine risk stratification within each molecular subgroup and assist clinicians in optimizing individualized treatment decisions. Nevertheless, without subgroup-specific verification data, we acknowledge that the predictive efficacy of our signature may differ across distinct CRC molecular subtypes, which constitutes an important limitation of this research.

Deep learning greatly improves the accuracy and efficiency of CRC pathological diagnosis. CNN and vision transformer models automatically analyze histopathological slides to identify tumors, grade lesions and predict molecular traits such as MSI status, reducing manual diagnostic bias. Unlike these image-focused tools, our GAT graph neural network mines multi-omics epigenetic and immune interactions. Overall, image-based deep learning aids pathological screening, while our omics-based GAT refines prognosis and target discovery. These two deep learning approaches complement each other to advance precise CRC diagnosis and treatment.

Notwithstanding these shortcomings, our results offer clear and systematic guidance for follow-up research. Prospective clinical cohorts are required to validate the prognostic performance of the core gene signature and the therapeutic efficacy of candidate drugs. Additional functional assays will help confirm the biological functions of core genes and their mechanisms underlying drug targeting. Incorporating spatial transcriptomics data can further clarify the spatial distribution of core genes and their crosstalk with immune cells in the TIME. Future studies may also explore the interplay between histone reader regulation and other epigenetic pathways, and develop targeted inhibitors against histone modification-related genes to promote precision therapy for CRC.

## 5. Conclusions

Integrating TCGA multi-omics data with deep learning algorithms, we systematically identified core and auxiliary genes associated with CRC. Based on the functions of these genes, we established a tiered drug recommendation strategy for CRC. Sapitinib, which targets CUL7, MYLIP and LAP3, was selected as the top-priority agent. It concurrently suppresses core oncogenic drivers and abnormal metabolism, two key pathological features of CRC. PF-4708671 exhibits high affinity for WWTR1 and CYP51A1, with potent activity against cancer stemness and lipid metabolic reprogramming, making it suitable for metastatic and drug-resistant CRC. As a first-line chemotherapeutic backbone, oxaliplatin newly targets SLC25A5 and *TNFRSF17* in addition to its canonical DNA-damaging effects, broadening its therapeutic mechanisms in CRC.

## Data Availability

The datasets analyzed in this study were obtained from publicly available databases, including TCGA, GSE39582, GSE132257, UniProtKB, STRING, hTFtarget, miRTarBase, RCSB PDB and PubChem. Detailed accession numbers, official URLs and data acquisition procedures are provided in the Materials and Methods section of this manuscript. All used datasets conform to the user access regulations of each public repository.

## Supplementary Materials

The following supporting information can be downloaded at: https://www.mdpi.com/article/10.3390/genes17080848/s1, Figure S1: Correlation of integrated functional similarity scores under three weighting schemes. (**A**) 0.6:0.4 versus 0.5:0.5. (**B**) 0.6:0.4 versus 0.4:0.6. (**C**) 0.5:0.5 versus 0.4:0.6. Each dot represents a single gene pair; Figure S2: Loss curve during GAT model training; Figure S3: Correlation between cosine similarity of GAT gene embeddings and original network edge weights. X-axis, original edge weight; Y-axis, cosine similarity of gene embeddings; Figure S4: t-SNE clustering of gene embedding profiles; Figure S5: C-index comparison of the RSF model between training and test cohorts; Figure S6: Boxplot of 5-fold cross-validation AUC values for the LightGBM model; Figure S7: ROC curve of the optimal fold from LightGBM cross-validation (AUC = 0.6806); Figure S8: Correlation of feature importance scores between LightGBM and RSF models; Figure S9: Ranked importance of integrated multi-omics features; Figure S10: Summary distribution of SHAP values across all features. Each dot denotes one sample. *x*-axis, SHAP value (positive values increase prognostic risk; negative values reduce risk); Y-axis, features ranked by overall importance. Color gradient indicates original feature expression level (red, high; blue, low); Figure S11: SHAP importance ranking of the top 10 predictive genes; Figure S12: Kaplan–Meier survival curves of 12 histone modification reader genes. Blue curves, high expression; orange curves, low expression; shaded regions, 95% CIs. X-axis, survival time; Y-axis, survival probability. All genes showed marginally significant log-rank *p*-values (0.05 < *p* < 0.10); Figure S13: PCA of tumor and normal samples in the GSE39582 dataset; Figure S14: GO functional enrichment of genes in the dark-green subnetwork; Figure S15: KEGG pathway enrichment of genes in the dark-green subnetwork; Figure S16: GO functional enrichment of genes in the light-purple subnetwork; Figure S17: KEGG pathway enrichment of genes in the light-purple subnetwork; Figure S18: GO functional enrichment of genes in the purple-red subnetwork; Figure S19: KEGG pathway enrichment of genes in the purple-red subnetwork; Figure S20: GO functional enrichment of genes in the cyan subnetwork; Figure S21: KEGG pathway enrichment of genes in the cyan subnetwork; Table S1: List of genes encoding histone modification readers; Table S2: List of differentially expressed histone modification reader genes; Table S3: All molecular docking result; Table S4: Top 10 molecular docking pairs ranked by binding energy; Table S5: Optimal molecular docking results for core target genes.
