# Supplementary material for: Graph-Based Multi-Omics Integration Reveals Prognostic Histone Modification Reader Genes and Candidate Drug Targets in Colorectal Cancer
Source: Genes (Basel). 2026 Jul 23;17(8):848. doi: 10.3390/genes17080848 (PMC13512344; doi:10.3390/genes17080848)
Supplement: Supplementary file 1 [file genes-17-00848-s001.zip › Captions.pdf]

**Supplementary Figures**

**Figure S1** Correlation of integrated functional similarity scores under three weighting schemes. (A) 0.6:0.4 versus 0.5:0.5. (B) 0.6:0.4 versus 0.4:0.6. (C) 0.5:0.5 versus 0.4:0.6. Each dot represents a single gene pair.

**Figure S2** Loss curve during GAT model training.

**Figure S3** Correlation between cosine similarity of GAT gene embeddings and original network edge weights. X-axis, original edge weight; Y-axis, cosine similarity of gene embeddings.

**Figure S4** t-SNE clustering of gene embedding profiles.

**Figure S5** C-index comparison of the RSF model between training and test cohorts.

**Figure S6** Boxplot of 5-fold cross-validation AUC values for the LightGBM model.

**Figure S7** ROC curve of the optimal fold from LightGBM cross-validation (AUC = 0.6806).

**Figure S8** Correlation of feature importance scores between LightGBM and RSF models.

**Figure S9** Ranked importance of integrated multi-omics features.

**Figure S10** Summary distribution of SHAP values across all features. Each dot denotes one sample. X-axis, SHAP value (positive values increase prognostic risk; negative values reduce risk); Y-axis, features ranked by overall importance. Colour gradient indicates original feature expression level (red, high; blue, low).

**Figure S11** SHAP importance ranking of the top 10 predictive genes.

**Figure S12** Kaplan–Meier survival curves of 12 histone modification reader genes. Blue curves, high expression; orange curves, low expression; shaded regions, 95% CIs. X-axis, survival time; Y-axis, survival probability. All genes showed marginally significant log-rank P-values ($0.05 < P < 0.10$).

**Figure S13** PCA of tumour and normal samples in the GSE39582 dataset.

**Figure S14** GO functional enrichment of genes in the dark-green subnetwork.

**Figure S15** KEGG pathway enrichment of genes in the dark-green subnetwork.

**Figure S16** GO functional enrichment of genes in the light-purple subnetwork.

**Figure S17** KEGG pathway enrichment of genes in the light-purple subnetwork.

**Figure S18** GO functional enrichment of genes in the purple-red subnetwork.

**Figure S19** KEGG pathway enrichment of genes in the purple-red subnetwork.

**Figure S20** GO functional enrichment of genes in the cyan subnetwork.

**Figure S21** KEGG pathway enrichment of genes in the cyan subnetwork.

**Supplementary Tables**

**Table S1** List of genes encoding histone modification readers.

**Table S2** List of differentially expressed histone modification reader genes.

**Table S3** All molecular docking result.

**Table S4** Top 10 molecular docking pairs ranked by binding energy.

**Table S5** Optimal molecular docking results for core target genes.
